# *Lyg1* deficiency aggravated LPS-induced chronic epididymal inflammation and sperm dysfunction in mouse

**DOI:** 10.3389/fimmu.2025.1699581

**Published:** 2025-12-09

**Authors:** XueXia Liu, YuXiao Zhang, JiaLu Wei, BenJiao Gong, XiaoXin Wang, FuJun Liu

**Affiliations:** 1 Shandong Stem Cell Engineering Technology Research Center, Affiliated Yantai Yuhuangding Hospital of Qingdao University, Yantai, China; 2 School of Bioscience and Technology, Shandong Second Medical University, Weifang, China

**Keywords:** LYG1, chronic epididymitis, fibrosis, sperm function, male fertility

## Abstract

**Introduction:**

Chronic epididymitis threatens male fertility, yet the role of LYG1 in this disease is unclear. LYG1, an immunomodulatory protein, is highly expressed in the mouse epididymis, especially the cauda region prone to fibrosis. This study aimed to explore whether Lyg1 deficiency exacerbates LPS-induced epididymal inflammation, fibrosis and sperm dysfunction.

**Methods:**

Sixty-four wild-type (WT) and Lyg1 knockout (KO) mice were divided into four groups. LPS was used to induce chronic epididymitis, with PBS as control. After 42 days, histological staining, flow cytometry, oxidative stress detection, transcriptomic profiling, sperm function analysis and In Vitro Fertilization (IVF) were performed to evaluate related indexes.

**Results:**

Under physiological conditions, Lyg1 KO mice had normal reproductive phenotypes. However, LPS-induced inflammation led to more severe epididymal damage, enhanced oxidative stress, abnormal ECM pathways and worse sperm dysfunction (reduced acrosomal integrity and functional proteins) in KO mice. IVF showed a more significant decrease in fertilization rate in KO mice.

**Discussion:**

LYG1 regulates the balance between epididymal inflammation and tissue repair. Its deficiency aggravates LPS-induced epididymal fibrosis and sperm dysfunction. This study identifies LYG1-dependent pathways as potential therapeutic targets for chronic epididymitis-related infertility.

## 1 Introduction

Chronic epididymitis, a common inflammatory disorder of the male reproductive tract, poses a substantial threat to male fertility by disrupting sperm maturation, impairing the epididymal microenvironment, and inducing fibrotic damage (1). Epidemiological studies indicate that up to 6% of men of reproductive age experience recurrent or persistent epididymitis, with chronic cases frequently associated with subfertility or infertility due to compromised sperm quality and transport dysfunction (2). The epididymis, a highly specialized coiled tubular organ essential for sperm maturation, acquisition of motility, and functional competence, depends on a delicate balance of immune tolerance, epithelial secretory activity, and extracellular matrix (ECM) homeostasis. Prolonged inflammation, such as that caused by bacterial infection, autoimmunity, or unresolved acute episodes, disrupts this balance, leading to epithelial damage, immune cell infiltration, and fibrotic remodeling, particularly in the cauda epididymis, where sperm undergo final maturation and storage (3). Although the role of proinflammatory cytokines (e.g., IL-6, TNF-α) and immune cell subsets in epididymal inflammation has been extensively documented (4), the molecular mechanisms underlying tissue resilience and pathological fibrosis in chronic epididymitis remain poorly understood.

Our group previous identified Lysozyme G-type 1 (LYG1) is a secreted protein with cytokine-like features (5). LYG1 shows tissue-specific patterns, with significantly high levels in immune-related organs such as the spleen, lymph nodes, bone marrow and epididymis (5). It is an important immunomodulatory protein that enhances the production of interferon-gamma (IFN-γ) by tumor antigen-specific CD4^+^ T cells, which in turn strengthens the cytotoxic function of CD8^+^ T cells, ultimately inhibiting tumor growth (5). This critical role is further supported by the observation that *Lyg1*-deficient mice exhibit accelerated tumor progression, accompanied by reduced T cell numbers and impaired IFN-γ production.​ LYG1 deficiency also attenuates the severity of acute graft-versus-host disease (aGVHD) by skewing allogeneic T cells toward regulatory T cells (Tregs), along with decreased expression of activation markers (e.g., CD69) and pro-inflammatory cytokines (e.g., IFN-γ) in T cells (6). These findings indicate that LYG1 is a pivotal molecule bridging innate and adaptive immunity, with significant therapeutic potential in modulating immune-mediated diseases. However, the expression and function of LYG1 in non-immune tissues, particularly in the male reproductive tract, remain largely uncharacterized. Given the high expression of Lyg1 in the mouse epididymis, especially in the cauda, a region prone to inflammatory fibrosis (7, 8), we proposed that LYG1 may play a critical role in maintaining epididymal epithelial integrity and immune homeostasis, thereby safeguarding sperm maturation under inflammatory stress.

Notably, lipopolysaccharide (LPS)-induced models of mouse epididymitis replicated key features of human chronic disease, such as cytokine upregulation, immune cell infiltration, and fibrotic remodeling. Thus, they served as valuable tools for mechanistic investigations (9). By integrating genetic knockout models, transcriptomic profiling, and functional assays, we aimed to examine whether *Lyg1* deficiency exacerbates LPS-induced epididymal inflammation and fibrosis, and how this effects sperm quality and fertilization capacity. Here, we reported that LYG1 deficiency exacerbated LPS-induced pathological alterations in the cauda epididymis. These changes included increased collagen deposition and epithelial disorganization in cauda epididymis, accompanied by reduced expression of sperm functional proteins critical for acrosomal integrity and motility. Transcriptomic analysis uncovered distinct molecular pathways in wild-type (WT) and *Lyg1* knockout (KO) mice. These findings identified LYG1as a novel mediator regulating the balance between epididymal inflammatory response and tissue repair. By addressing the unmet need to understand the genetic factors contributing to inflammatory epididymal dysfunction, our study offered insights into potential therapeutic targets for alleviating fibrosis and preserving male fertility in chronic epididymitis.

## 2 Methods

### 2.1 Animals and grouping

Six-week-old male C57BL/6 mice, weighing approximately 20–22 g, were obtained from Beijing Vital River Laboratory Animal Technology Co., Ltd. The *Lyg1* conventional knockout mice (on a C57BL/6 background, *Lyg1^-/-^)* were kindly donated by Prof. Wenling Han at Peking University Health Science Center (Beijing, China). All mice were bred in a specific pathogen-free (SPF) facility. The environmental conditions were maintained at a stable temperature of 22 ± 2 °C, a relative humidity of 45 ± 5%, and a 12-hour light/dark cycle. Throughout the study, all mice had unrestricted access to food and water. All experimental procedures performed in this study were approved by the Medical Ethics Committee of Yantai Yuhuangding Hospital (2025-222).

Sixty-four mice were randomly divided into four groups: the PBS-treated control group and the LPS-treated experimental group, which included both WT mice and Lyg1 KO mice. Specifically, in the preliminary experiment, four mice were randomly assigned to each group for sample collection to assess cytokine expression 72 hours post-treatment, thereby confirming the successful construction of the mouse model. In the main experiment, the remaining 12 mice in each group were kept until day 42 to observe the effects on the epididymis and sperm function (**Supplementary Figure 1**). The control group received an intraperitoneal injection of PBS, while the LPS-treated group received an intraperitoneal injection of LPS (HY-D1056, MedChemExpress LLC, Shanghai, China). All mice were anesthetized via intraperitoneal injection of 2,2,2-Tribromoethanol (240 mg/kg, Avertin, AbMole, USA; catalog number M14641) and euthanized by cervical dislocation when necessary. These procedures were performed 42 days post-treatment for subsequent analyses. One epididymis from each mouse was collected and fixed in Bouin’s solution (HT10132, Sigma, St. Louis, MO, USA) for immunohistochemical staining and microscopic evaluation. The contralateral epididymis was rapidly frozen in liquid nitrogen for subsequent mRNA and protein extraction. Epididymal spermatozoa were collected from the cauda epididymis and suspended in PBS supplemented with 10% (w/v) bovine serum albumin (BSA). Several small incisions were made to release live sperm at 37°C for 5 minutes. Sperm parameters were quantified using a Computer-Assisted Sperm Analysis (CASA) system (Hamilton Thorne, Beverly, MA, USA).

### 2.2 Histological and indirect immunofluorescence analysis

Hematoxylin and eosin (HE) staining was performed according to a previously established protocol to assess morphological alterations (10). Paraffin-embedded sections were deparaffinized through three cycles of xylene treatment, followed by rehydration using a graded ethanol series decreasing from 100% to 75%. The sections were subsequently stained with hematoxylin for 10 minutes and rinsed under running water for 2 minutes. Eosin staining was then applied for 1 minute. After dehydration, the slides were mounted and examined under light microscopy (Axio Scope5, Carl Zeiss Jen, Germany). For Masson’s trichrome staining, tissue sections were stained with hematoxylin for 5 minutes, followed by rinsing in distilled water for 5 minutes. Differentiation was achieved using 1% acid alcohol for 30 seconds, and the sections were washed under running tap water for 10 minutes. The sections were then incubated with Ponceau S-acid fuchsin solution (50 μL/section) for 10 minutes, differentiated with 1% phosphomolybdic acid for 2 minutes, and stained with 0.5% aniline blue (50 μL/section) for 1 minute. After rinsing in distilled water, the sections were differentiated with 1% acetic acid for 1 minute. Following dehydration, the slides were mounted and examined under light microscopy (Axio Scope5, Carl Zeiss Jen, Germany). For each experimental group, five mice were used for HE staining and Masson’s trichrome staining. For each mouse, 10 random fields were imaged at 400× magnification.

For IF analysis, deparaffinized slides were subjected to antigen retrieval by heating in a citrate buffer solution (0.01 M, pH 6.0) at medium power in a microwave for 20 minutes. Endogenous peroxidase activity was inhibited using 3% hydrogen peroxide (v/v), and non-specific protein binding sites were blocked with Tri-buffered saline (TBS) containing 3% (w/v) BSA at room temperature for one hour. Subsequently, the slides were incubated overnight at 4 °C with primary antibody (Anti-CD45, DF6839; Anti-NRF2, AF7006; Anti-HO-1, AF5393; Affinity Biosciences, JiangSu, China). After three washes with TBS, the slides were incubated at 37°C for one hour with FITC-labeled anti-rabbit IgG secondary antibody diluted at a ratio of 1:400. Nuclei was counterstained with propidium iodide (PI; Invitrogen Carlsbad CA; concentration: 0.01 mg/ml). Images were captured using a fluorescent microscopy (Observer7 Carl Zeiss Jena Germany). The average number of positive-staining signals was quantified and calculated using ImageJ software. Immunofluorescence intensity of each protein was expressed as the ratio of fluorescent signal intensity (FITC) to lumen area, with at least 20 lumens analyzed for positive signals. IF images of CD45 staining cells were captured at 400× magnification. For each mouse (*n* = 3 per group), 8 non-overlapping fields of view (FOV) were selected from both caput and cauda epididymis, avoiding tissue edges. The numerical density (Nv) was determined as the total number of positive stained cells per FOV divided by the area of the FOV (mm²), expressed as cells/mm². Data were reported as mean ± SD of Nv values across all FOVs for each group. For sperm IF quantitative analysis, spermatozoa were fixed onto gelatin-coated coverslips (1% gelatin) and treated with cold methanol for 10 minutes for fixation. Non-specific binding was blocked with 3% BSA in TBS at room temperature for one hour. The samples were then incubated overnight at 4°C with primary antibodies (anti-TSSK2, ab192026; anti-ZPBP, ab97691; anti-HSPA4L, DF2503; all from Abcam, Cambridge, UK, or Affinity Biosciences, JiangSu, China). Following three washes with PBS, the samples were incubated with FITC-labeled anti-rabbit IgG secondary antibody at room temperature for one hour. Pre-immune IgG was used as a negative control. Nuclei were counterstained with PI. Sections were mounted in 80% glycerol and examined under a fluorescence microscope (Observer 7, Carl Zeiss Jena, Germany) according to our previously published methods (11). Briefly, at least 200 spermatozoa per mouse were counted across multiple fields of view. The localization rate of positive sperm was calculated as the number of sperm expressing the target protein divided by the total counted sperm. The fluorescence intensity of the sperm protein was quantified as the ratio of its fluorescence intensity to the PI fluorescence intensity in the sperm.

### 2.3 Oxidative stress indicators detection

Oxidative stress markers of superoxide dismutase (SOD, S0103, Beyotime, China) and malondialdehyde (MDA, S0131S, Beyotime, China) were detected in mice cauda epididymis homogenate. The tissues were extracted and measured according to manufacturer’s instruction. Optical density (OD) values were read using microplate reader (Varioskan, Thermo Scientific ShangHai, China) and analyzed by ImageJ.

### 2.4 Flow cytometry for specific immune cell subsets in cauda epididymis

Cauda epididymis tissues were dissected from mice 42 days post-treatment and minced into small pieces (1–2 mm³) in cold phosphate-buffered saline (PBS) containing 1% bovine serum albumin (BSA). The tissue fragments were digested with 0.25% trypsin-EDTA (Gibco, USA) at 37°C for 30 minutes with gentle shaking. The digestion was terminated by adding an equal volume of RPMI 1640 medium (Gibco, USA) supplemented with 10% fetal bovine serum (FBS, Gibco, USA). The cell suspension was filtered through a 70-μm cell strainer (BD Biosciences, USA) to remove undigested tissue debris, followed by centrifugation at 300×g for 5 minutes at 4°C. The cell pellet was resuspended in cold PBS with 1% BSA, and the total cell number was counted using a hemocytometer. For surface marker staining, 1×10^6^ cells per sample were incubated with fluorochrome-conjugated primary antibodies for 30 minutes at 4°C in the dark. The antibodies used included anti-mouse CD45-PE (clone 30-F11, BD Biosciences), anti-mouse F4/80-FITC (clone BM8, eBioscience), and anti-mouse CD86-APC (clone GL-1, eBioscience) for M1 macrophage identification. Isotype-matched control antibodies (BD Biosciences) were used to set the gating threshold. After staining, cells were washed twice with cold PBS containing 1% BSA and resuspended in 300 μL of PBS for flow cytometric analysis. Flow cytometry was performed using a BD FACSCanto II flow cytometer (BD Biosciences, USA), and data were analyzed with FlowJo software (version 10.8.1, Tree Star). Gating strategies were established to exclude dead cells and debris based on forward scatter (FSC) and side scatter (SSC). CD45^+^ cells were identified as leukocytes, F4/80^+^ cells as macrophages, and CD45^+^F4/80^+^CD86^+^ cells as M1 macrophages. The proportion of each immune cell subset was calculated relative to the total number of viable cells. All experiments were performed in triplicate, and results are presented as mean ± standard deviation (SD).

### 2.5 RNA extraction and RT-PCR

Total RNA was extracted from each sample using TRIzol reagent according to the protocols described in our previous publication (12). Subsequently, cDNA synthesis was performed using the 5 × All-In-One RT MasterMix with AccuRT (G592, Abm, Jiangsu, China), strictly following the manufacturer’s instructions. Quantitative PCR reactions were conducted using BlasTaq 2 × qPCR MasterMix (G891, Abm, Jiangsu, China) on an ABI Prism 7500 instrument (Thermo Scientific, Shanghai, China). Primer sequences are listed in **Supplementary Table 1**. *Actb* was used as the internal reference gene. Cycle threshold (CT) values for each target gene were recorded, and relative expression levels were calculated using the 2^-ΔΔCT^ method. Statistical analysis was performed using One-Way ANOVA.

### 2.6 Transcriptomic profiling

RNA-Seq libraries were constructed from cauda epididymidis tissues using the TruSeq Stranded mRNA Library Prep Kit (Illumina) and sequenced on an Illumina NovaSeq 6000 platform. Differential gene expression analysis was performed using the DESeq2 in R. Gene Ontology (GO) enrichment and Kyoto Encyclopeida of Genes and Genomes (KEGG) pathway analyses were conducted using the DAVID Functional Annotation Bioinformatics Microarray Analysis tools (https://davidbioinformatics.nih.gov).

### 2.7 Sperm function analysis

Sperm motility and count were assessed using CASA. Acrosomal integrity was evaluated by PSA staining (FITC-PSA, L0770, Sigma, St. Louis, MO, USA). Briefly, sperm smears were stained with FITC-PSA for 30 min, and the nuclei were counterstained with PI. The samples were then washed with PBS before being observed under fluorescence microscope (Observer 7, Carl Zeiss Jena, Germany). On average, 200 spermatozoa were analyzed per slide. The localization rate of positive sperm was calculated as the number of sperm with positive PSA staining divided by the total counted sperm.

### 2.8 *In vitro* fertilization

Female mice were induced to undergo superovulation via intraperitoneal injection of 5 IU pregnant mare serum gonadotropin (PMSG) and human chorionic gonadotropin (hCG), administered at 48-hour intervals. Cumulus-oocyte complexes (COCs) were harvested 14–17 hours post-hCG injection. Following euthanasia, the COCs were transferred to 200 μL droplets of HTF medium (72002, SUDGEN, Nanjing, China), which were incubated under conditions of 37°C, saturated humidity, and 5% CO_2_. Epididymal sperm from male mice (2 × 10^5^ sperm per group) were collected from the cauda epididymis and capacitated in 200 μL of C-TYH medium (72021, SUDGEN, Nanjing, China) for 30 minutes at 37°C in a 5% CO_2_ incubator. Subsequently, approximately 10^4^ sperm were transferred into pre-equilibrated HTF medium (72002, SUDGEN, Nanjing, China). After a 4-hour incubation period, fertilized oocytes were washed and transferred to KSOM medium (M1430, AIBEI, Nanjing, China).

### 2.9 Statistical analysis

The data were presented as the mean ± standard deviation from three independent replicates. Normality was assessed using the Anderson-Darling test, and significant differences among groups were analyzed via One-Way ANOVA followed by Tukey’s *post-hoc* test for multiple comparisons. All statistical analyses were performed using GraphPad Prism version 9 (GraphPad Software, La Jolla, CA, USA). A *p*-value of less than 0.05 was considered statistically significant.

## 3 Results

### 3.1 Expression characteristics of *Lyg1* in mouse reproductive tissues

**Figure 1:**
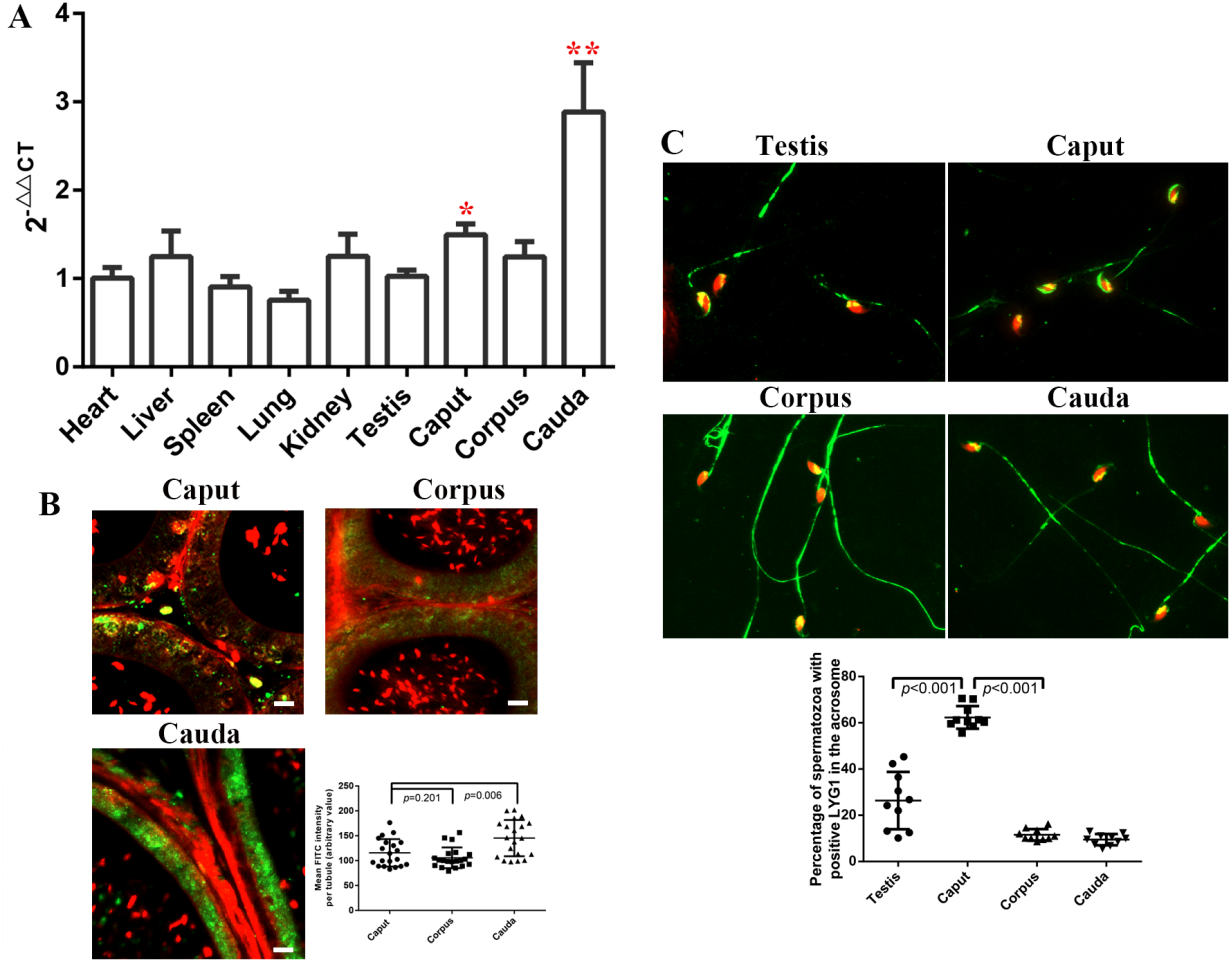
The expression of *Lyg1* in various mouse tissues and its localization within mouse sperm **(A)** mRNA expression of *Lyg1* in different mouse tissues (n=4); **(B)** Localization of LYG1 in caput, corpus and cauda mice epididymis; **(C)** LYG1 expression in mouse sperm from the testis, caput, corpus and cauda epididymis. The statistical analysis was performed by One-Way ANOVA, and p value less than 0.05 was considered significance. * indicated p<0.05, and ** indicated p<0.01.

RT-PCR analysis revealed significantly higher expression levels of *Lyg1* in the epididymis, particularly in the cauda epididymidis, compared with the testis and other reproductive tissues (**Figure 1A**). Immunofluorescence staining further confirmed the high expression of LYG1 in the cauda epididymidis (**Figure 1B**). Subsequently, we analyzed the localization of LYG1 in sperm. The results showed that LYG1 was primarily localized to three regions of sperm: the acrosome, the posterior acrosomal region, and the middle piece of the sperm. Notably, the localization of LYG1 in the acrosome was significantly more abundant in testicular sperm (26.35 ± 12.40) and caput epididymal sperm (62.33 ± 4.90) than in other sperm populations (corpus: 11.57 ± 2.46; cauda: 9.48 ± 2.41) (**Figure 1C**). These findings suggest that LYG1 plays a critical role in acrosome-related biological processes during sperm maturation, thereby implying its potential involvement in regulating sperm maturation and maintaining sperm structural integrity.

### 3.2 Characteristics of male fertility in *Lyg1* KO mice

**Figure 2:**
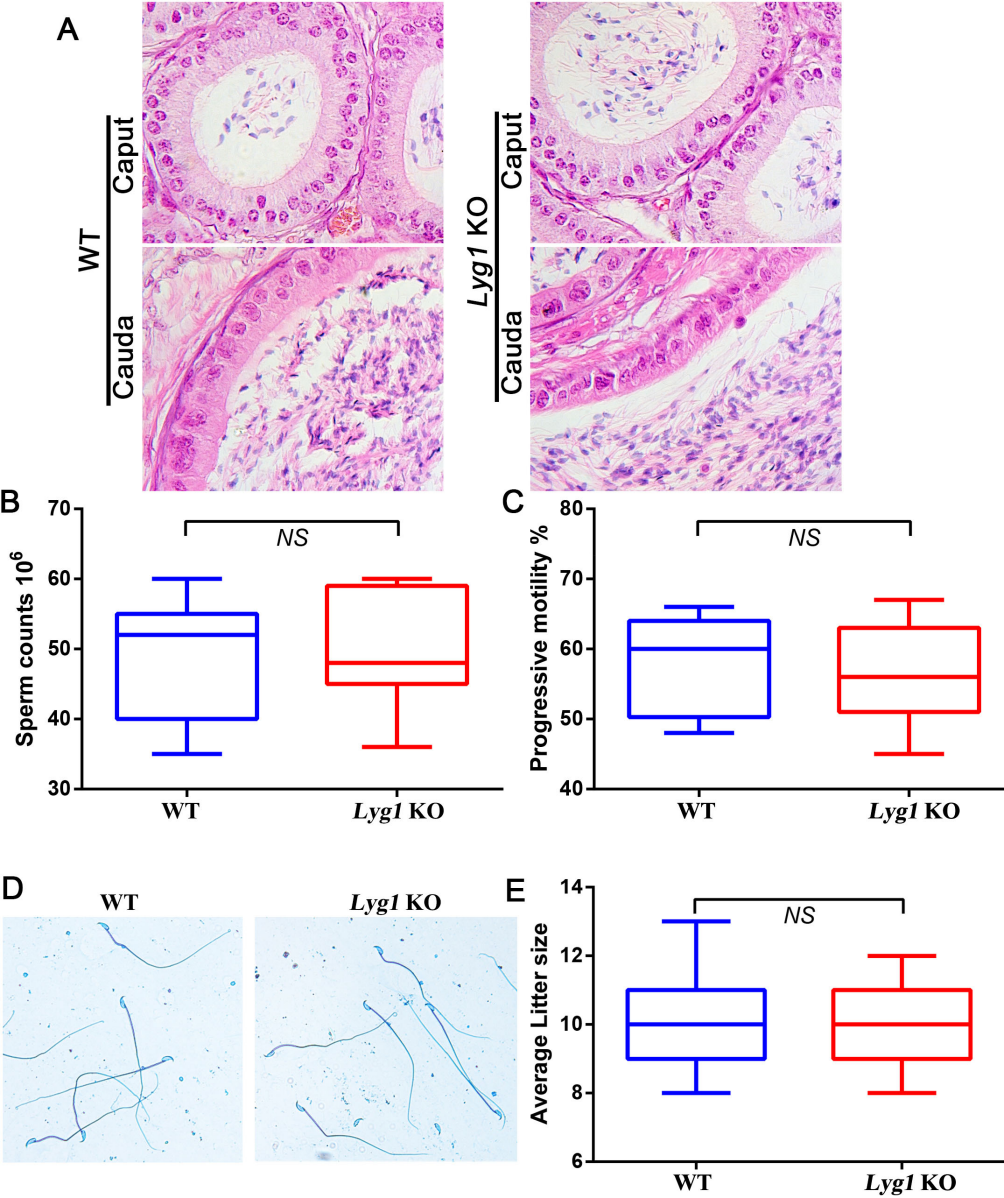
Morphological characteristics of the epididymis (caput and cauda regions), sperm concentration, motility parameters, and male fertility in WT and *Lyg1* KO mice **(A)** Epididymal morphology was assessed using HE staining; **(B, C)** Sperm counts and progressive motility were analyzed using CASA; **(D)** Sperm morphology was visualized using CBB staining; **(E)** Male fertility was evaluated based on average litter size. The statistical analysis was performed by *Student’s t test*, and p value less than 0.05 was considered significance. NS: No Significance.

To characterize the reproductive phenotypes of *Lyg1* KO mice, we comprehensively analyzed epididymal histology, sperm morphology, motility parameters, and male fertility (**Figure 2**). These analyses demonstrated no statistically significant disparities in epididymal histological features, sperm morphological traits, motility parameters, or male fertility metrics between WT and *Lyg1* KO mice. Collectively, these findings indicate that Lyg1 ablation does not appreciably impair male fertility under standard physiological conditions.

### 3.3Lyg1 deficiency exacerbates LPS-induced epididymal damage

**Figure 3:**
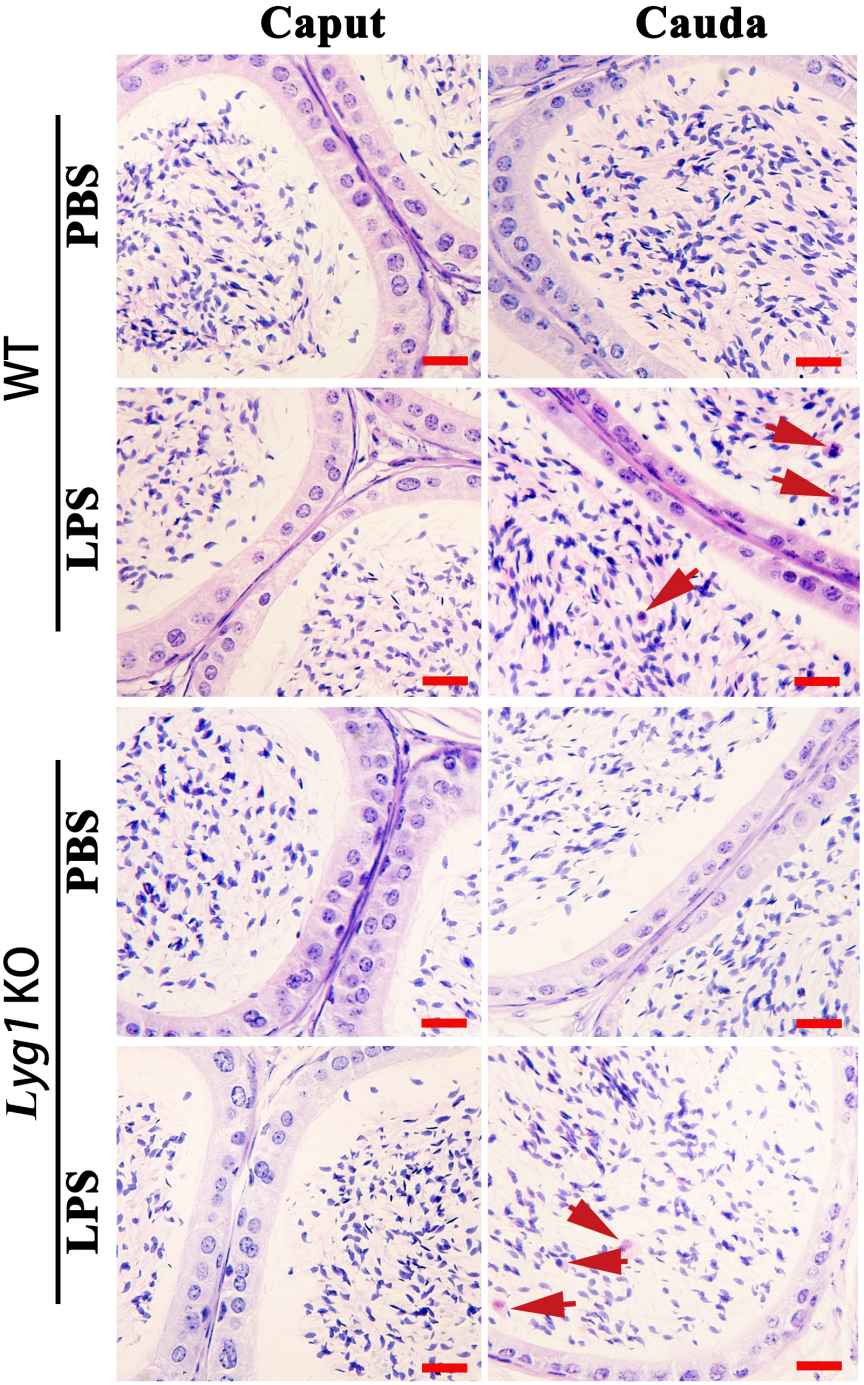
Morphological analysis PBS and LPS treated epididymis in WT and *Lyg1* KO mice. The red arrow indicated the inflammatory cells. Each bar represented 20 µm.

**Figure 4:**
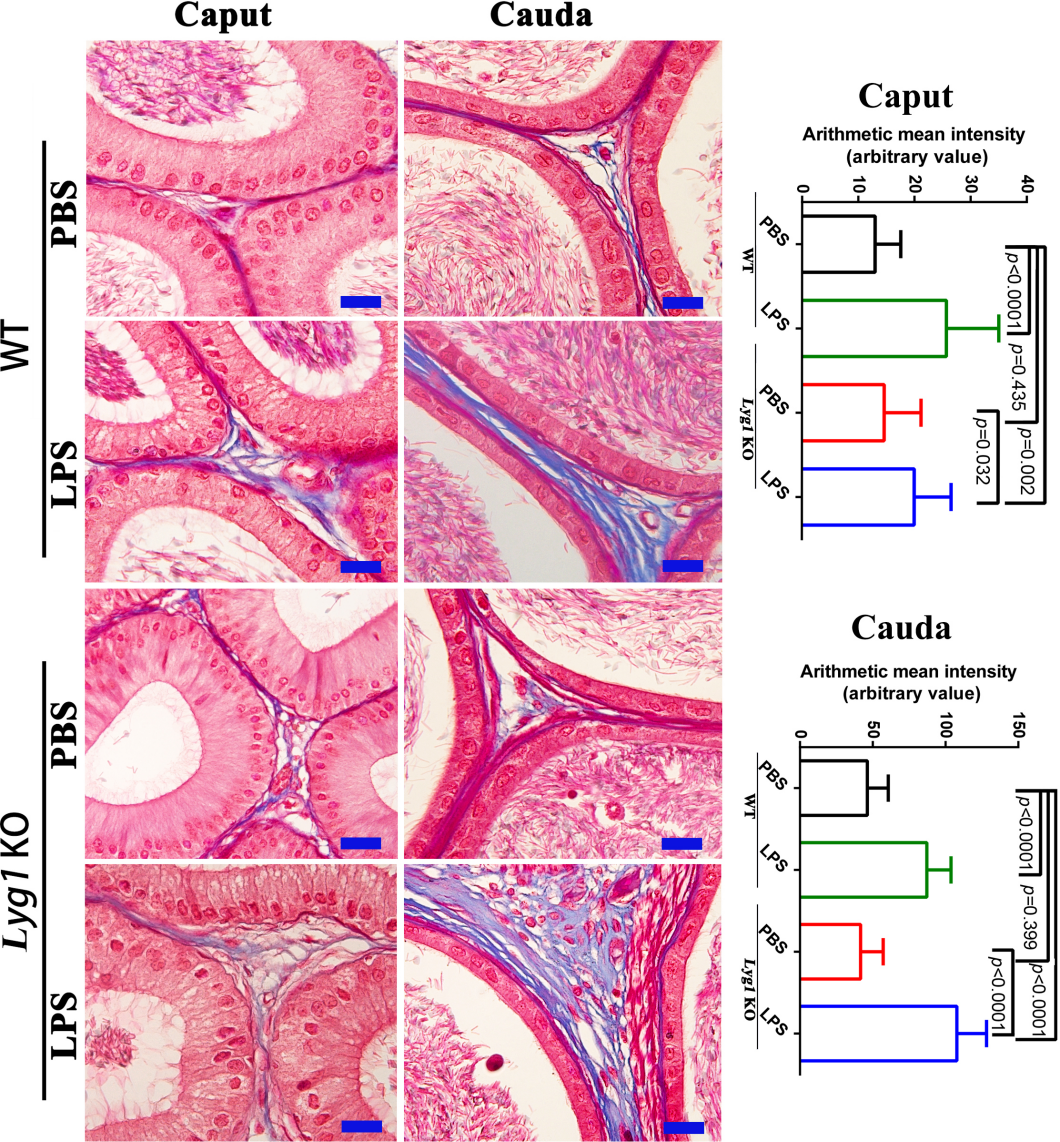
Masson staining analysis of collagen fibers in the caput and cauda epididymis from PBS and LPS treated mice. The statistical analysis was performed by One-Way ANOVA, and p value less than 0.05 was considered significance. Each bar represented 20 µm.

As LYG1 is a key inflammation-associated molecule, we aimed to establish a lipopolysaccharide (LPS)-induced epididymitis model to investigate whether LYG1 participates in the pathogenesis of epididymitis. The LPS-induced mouse epididymitis model was validated by significantly increased expression of IL-6 and TNF-α in both the caput and cauda epididymis at 72 hours post-injection (**Supplementary Figure 2**). Tissue samples were collected at 42 days post-injection to assess changes in epididymal structure and function. Hematoxylin and eosin (HE) staining at 42 days revealed mild epithelial disarray in the caput epididymis of mice from both groups. In contrast, severe inflammation, characterized by immune cell infiltration and epithelial disruption, was observed in the cauda epididymis of LPS-treated mice, with a more pronounced phenotype in Lyg1 KO mice (**Figure 3**). Masson’s trichrome staining further demonstrated enhanced collagen deposition in the cauda epididymis of Lyg1 KO mice, indicating exacerbated ECM fibrosis (**Figure 4**).

IF staining combined with quantitative analysis revealed significant differences in immune cell infiltration among the different treatment groups. Specifically, CD45^+^ leukocytes were primarily localized in the interstitial regions of the epididymis. Following LPS treatment: In WT mice, the number of CD45^+^ cells in the caput epididymis increased by 2.12-fold (PBS: 4.24 ± 0.46 vs. LPS: 9.00 ± 0.73), and by 3.68-fold in the cauda epididymis (PBS: 3.54 ± 0.50 vs. LPS: 13.04 ± 1.19). In Lyg1 KO mice, the number of CD45^+^ cells in the caput epididymis increased by 2.10-fold (PBS: 5.42 ± 0.62 vs. LPS: 11.38 ± 1.01), and by 4.89-fold in the cauda epididymis (PBS: 3.75 ± 0.34 vs. LPS: 18.33 ± 1.53) (**Supplementary Figure 3**).

Furthermore, flow cytometry was employed to analyze the distribution of immune cells in the cauda epididymis, and the results indicated that LPS treatment significantly increased the proportions of CD45^+^ leukocytes (WT:4.13 ± 0.31 vs. KO: 4.83 ± 0.42), F4/80^+^ immune cells (WT:1.17 ± 0.11 vs. KO: 1.41 ± 0.07), and M1 macrophages (WT:0.23 ± 0.02 vs. KO: 0.29 ± 0.04) in the cauda epididymis (**Supplementary Figure 4**). These findings collectively suggest that Lyg1 may play a regulatory role in LPS-induced immune cell infiltration and macrophage polarization in the epididymis, with its deficiency potentiating the inflammatory response.

### 3.4 Lyg1 deficiency enhances lps-induced oxidative stress via impairing NRF2-HO-1 pathway

To explore the role of LYG1 in oxidative stress regulation during epididymitis, we detected the activity of SOD and the content of MDA in cauda epididymis homogenates. Under physiological conditions (PBS treatment), there was no significant difference in SOD activity between WT and Lyg1 KO mice (p=0.439). However, LPS stimulation significantly reduced SOD activity in both genotypes, and the reduction was more pronounced in Lyg1 KO mice (p=0.044) (**Supplementary Figure 5**). Conversely, MDA levels, a marker of lipid peroxidation, were significantly elevated in LPS-treated KO mice compared to WT mice indicating exacerbated oxidative damage due to Lyg1 deficiency (**Supplementary Figure 5**).

Immunofluorescence analysis of NRF2 and its downstream target HO-1 (key regulators of oxidative stress response) revealed distinct expression patterns. LPS treatment significantly downregulated NRF2 and HO-1 expression in the cauda epididymis. Lyg1 KO mice showed a more downregulation expression of NRF2 (WT: 58.18 ± 5.61 vs. KO:50.15 ± 3.77) and HO-1 (WT: 66.68 ± 4.22 vs. KO:65.54 ± 4.11) after LPS exposure (**Supplementary Figure 6**). These results demonstrated that Lyg1 deficiency impairs the activation of the NRF2-HO-1 antioxidant pathway, thereby amplifying LPS-induced oxidative stress in the epididymis.

### 3.5 Transcriptomic profiling highlighted distinct pathways in WT and KO mice

Given the significant pathological, immunological, and oxidative stress disparities observed between WT and Lyg1 KO mice in response to LPS-induced epididymitis, we further performed transcriptomic profiling of cauda epididymis tissues to unravel the underlying molecular pathway differences that drive these phenotypic variations. RNA sequencing analysis of cauda epididymis tissues identified distinct differentially expressed genes (DEGs) in LPS-treated WT and *Lyg1* KO mice. In WT mice, 69 genes were significantly upregulated and 45 were significantly downregulated (fold change≥1.5, p<0.05) (**Supplementary Table 1**). LPS treatment induced the upregulation of immune- and metabolic-related genes (e.g., Retnla, Gapdhs) and ECM regulators (e.g., Sparc), while simultaneously downregulating genes involved in ubiquitination (e.g., Rnf5) and apoptosis (e.g., Gadd45a). In contrast, in *Lyg1* KO mice, 106 genes were significantly upregulated and 53 were significantly downregulated (**Supplementary Table 2**). KO mice exhibited marked enhanced upregulation of ECM-receptor interactions (e.g., Col6a3, Thbs1) and adhesion molecules (e.g., Acta1), along with the suppression of hormone signaling pathways (e.g., Vipr1) and detoxification mechanisms (e.g., Aldh3b2). KEGG pathway analysis revealed metabolic reprogramming and cytokine signaling as predominant features in WT mice, whereas fibrosis-associated pathways were enriched in KO mice. Notably, Pi16 (a protease inhibitor) was consistently upregulated in both groups, while Aldh3b2 (involved in oxidative stress response) was downregulated, suggesting conserved inflammatory pathways that depend on LYG1 (**Supplementary Table 3**, **Supplementary Figures 7**, **8**).

### 3.6 Lyg1 KO aggravated LPS-induced sperm dysfunction

**Figure 5:**
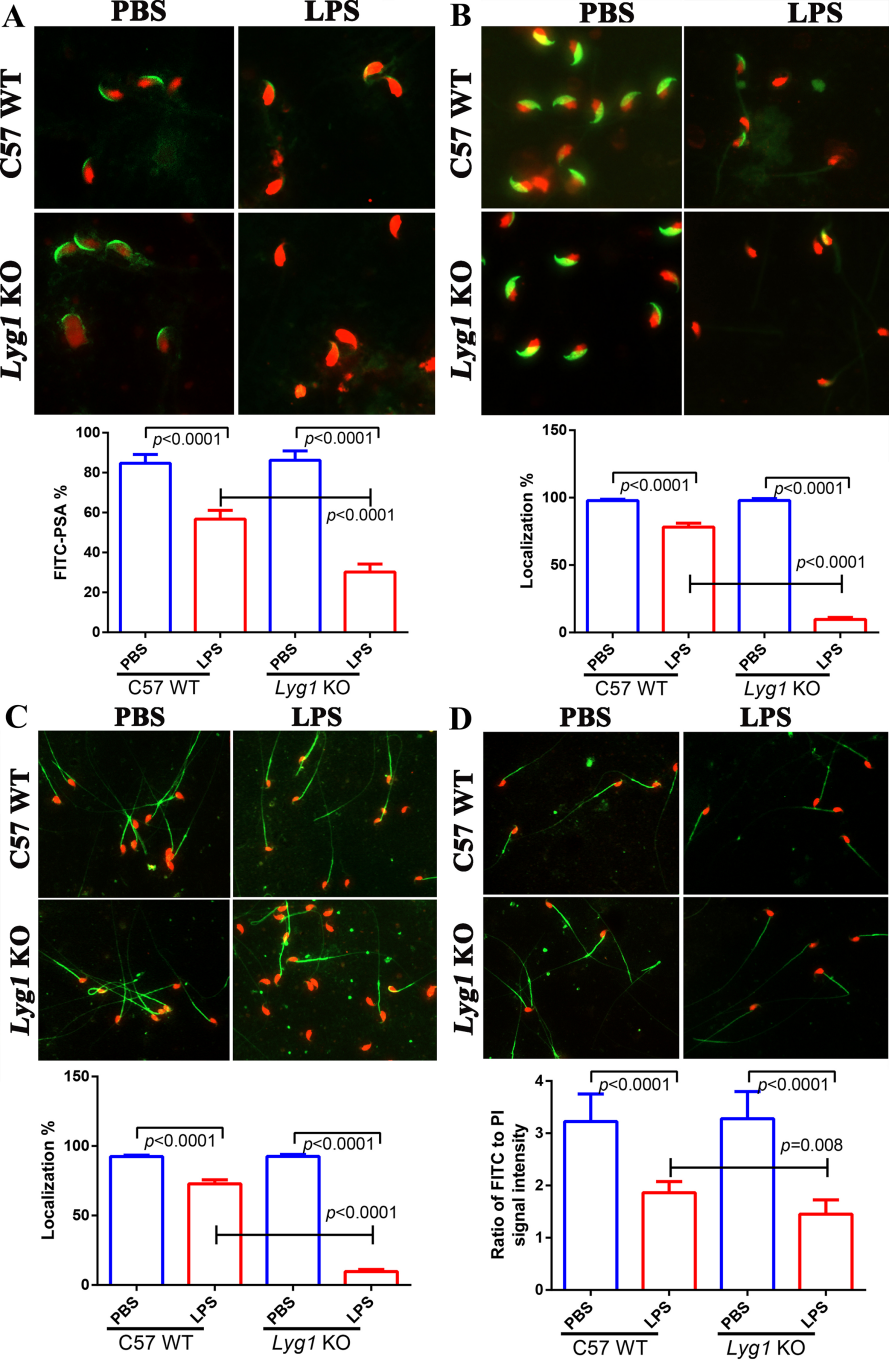
Immunofluorescence analysis of FITC-PSA, ZPBP、TSSK2、HSPA4L in spermatozoa from PBS and LPS-treated mice. **(A)** the percentage of spermatozoa stained with FITC-PSA; **(B)** the localization percentage of ZPBP in spermatozoa; **(C)** the localization percentage of TSSK2 in spermatozoa; **(D)** the intensity of HSPA4L in spermatozoa. The statistical analysis between two groups was performed by *Student’s t test*, and p value less than 0.05 was considered significance. *p* value less than 0.05 was considered as the significance.

LPS exposure significantly compromised acrosomal integrity and led to the downregulation of PSA positive staining and the decreased expression of sperm-specific proteins (ZPBP) in both groups. However, these effects were more pronounced in KO mice (**Figures 5A, B**). Furthermore, Both TSSK2 and HSPA4L were detected in the sperm tail, particularly in the middle segment. No significant difference was observed in the expression intensity of TSSK2 (**Figure 5C**). However, the localization rate of TSSK2 in sperm was significantly reduced in both the LPS-treated group and the KO group. In contrast, the localization rate of HSPA4L did not differ significantly among groups, although its expression intensity was markedly decreased following LPS treatment and exhibited a distinct pattern in the KO group compared to controls (**Figure 5D**).

### 3.7 Effects of LPS treatment on fertility in *Lyg1* KO and WT male mice

**Figure 6:**
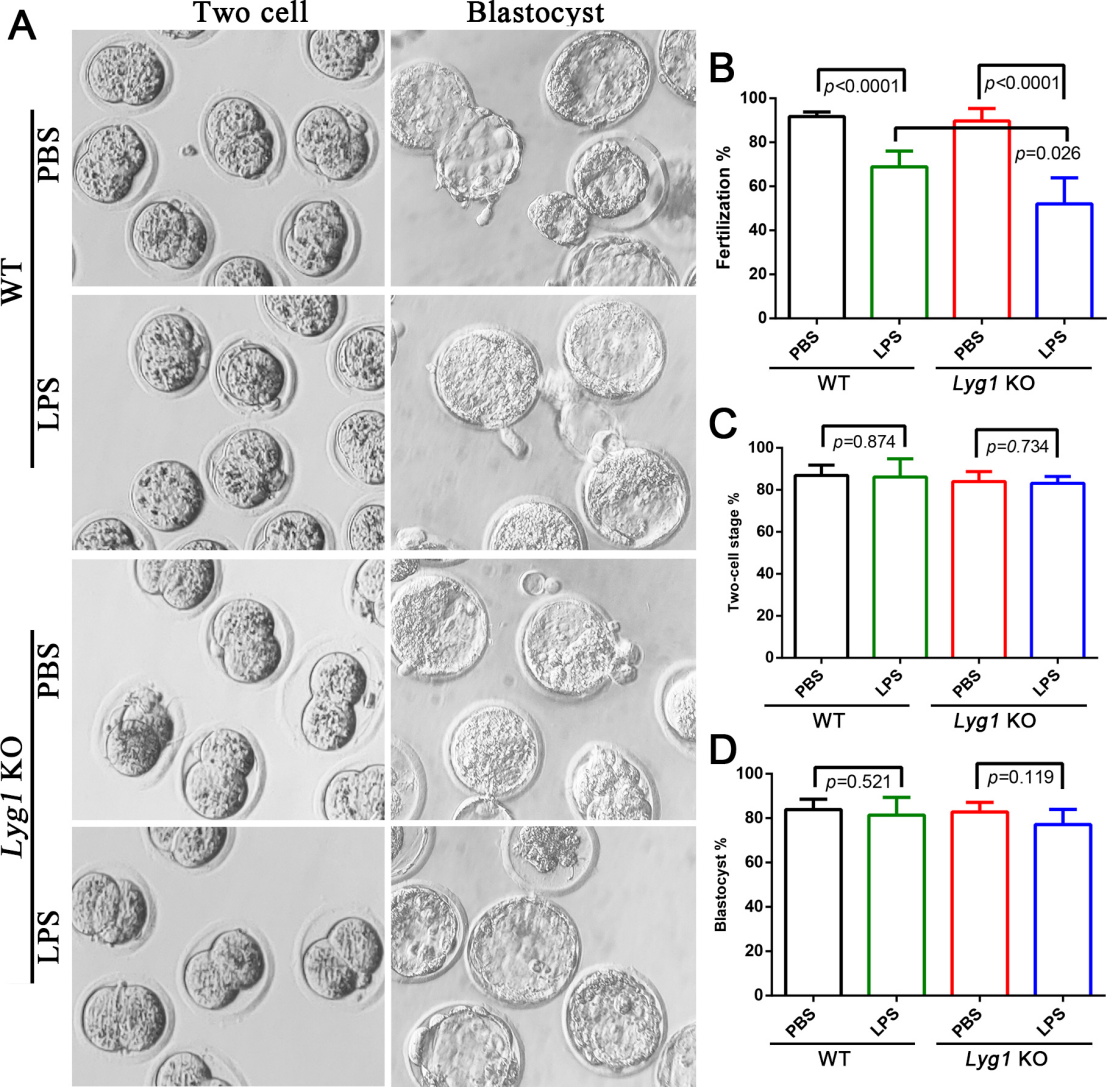
Characteristics of sperm fertility ability. **(A)** Representative image of IVF results; **(B)** Statistical analysis of the fertilization rate, defined as the percentage of oocytes exhibiting pronucleus formation relative to the total number of M II oocytes; **(C)** Statistical analysis of the two-cell development rate, defined as the percentage of two-cell embryos relative to the total number of oocytes with pronucleus formation; **(D)** Statistical analysis of the blastocyst development rate, defined as the percentage of blastocyst relative to two-cell embryos; The statistical analysis between two groups was performed by *Student’s t test*, and p value less than 0.05 was considered significance. *p* value less than 0.05 was considered as the significance.

IVF experiments were performed to assess the fertility of sperm from each experimental treatment group. The results demonstrated that LPS treatment markedly decreased the fertilization rates of both WT and *Lyg1* KO sperm, with a more pronounced reduction observed in *Lyg1* KO sperm. However, the formation rates of two-cell embryos and blastocyst derived from PBS-treated and LPS-treated KO mouse sperm did not exhibit any significant differences compared to the WT (**Figure 6**).

## 4 Discussion

Our findings delineate a critical role of LYG1 in orchestrating the balance between inflammatory resolution and tissue repair within the epididymis, a tissue essential for sperm maturation and functional competence. The region-specific high expression of Lyg1 in the cauda epididymis—where sperm undergo final maturation and storage—highlights its specialized adaptation to the unique microenvironmental demands of this segment. Under physiological conditions, Lyg1 KO mice exhibited unaltered epididymal histology, sperm parameters, and fertility, indicating that LYG1 is dispensable for basal reproductive homeostasis but becomes pivotal under inflammatory stress.

Upon LPS-induced chronic inflammation, WT mice mounted a coordinated adaptive response characterized by upregulation of immune resolution genes (e.g., Ccl8, Il33) and metabolic pathways (e.g., Gapdhs, Hk1). These pathways likely facilitate pathogen clearance while sustaining the energy-intensive processes of sperm maturation, underscoring a protective mechanism that preserves tissue function amid inflammation. In stark contrast, Lyg1 KO mice failed to activate this balanced response. Instead, transcriptomic profiling revealed enrichment of ECM-receptor interaction (e.g., Col6a3, Thbs1) and cell adhesion pathways (e.g., Acta1) in KO mice, accompanied by enhanced collagen deposition and epithelial disarray in the cauda epididymis. This dysregulated ECM remodeling likely disrupts the epithelial niche required for sperm-epithelial crosstalk, impairing sperm capacitation and rendering them vulnerable to oxidative stress.

The sperm-specific localization of LYG1 to the acrosome and middle piece provides a mechanistic link between epididymal inflammation and fertilization deficits. LPS-induced downregulation of acrosomal proteins ZPBP (critical for acrosome biogenesis) and TSSK2 (essential for tyrosine phosphorylation during capacitation) was more pronounced in KO sperm, which may compromise sperm-zona pellucida binding and the acrosome reaction (13, 14). Similarly, reduced expression intensity of the tail-specific chaperone HSPA4L, which is required for flagellar structure and motility (15), in LPS-treated KO sperm further supports LYG1’s role in protecting sperm proteins from inflammatory degradation or misfolding. Consistent with these molecular defects (12, 16), IVF experiments demonstrated a more severe reduction in fertilization rates in KO sperm, while early embryo development (two-cell and blastocyst formation) remained unaffected. This dissociation indicates that LYG1 primarily modulates pre-fertilization events (e.g., sperm motility, acrosomal integrity) rather than post-fertilization embryo viability, a distinction with clinical relevance for diagnosing male factor infertility.

Notably, our study extends the known functions of LYG1 beyond adaptive immunity. Previous reports identified LYG1 as a regulator of T cell activation in tumor immunity and acute graft-versus-host disease (aGVHD) (6). Here, we demonstrate for the first time that LYG1 modulates chronic inflammation-induced tissue fibrosis and sperm dysfunction, uncovering a novel role in reproductive tract homeostasis. Compared to existing chronic epididymitis research— which predominantly focuses on pro-inflammatory cytokines (e.g., IL-6, TNF-α) and immune cell infiltration—our work links immune regulation to ECM remodeling and sperm function, identifying LYG1 as a key mediator of this crosstalk.

The shared upregulation of Pi16 (a protease inhibitor) and downregulation of Aldh3b2 (involved in oxidative stress defense) in both WT and KO mice suggest conserved core inflammatory pathways independent of LYG1 (17, 18). However, the WT-specific enrichment of metabolic and immune resolution pathways versus KO-specific ECM overactivation highlights LYG1 as a “rheostat” that biases the inflammatory response toward resolution rather than fibrosis. This is particularly relevant to human chronic epididymitis, which is characterized by fibrotic scarring rather than acute infection (19), making the Lyg1 KO model a valuable tool for studying pathological fibrosis in male reproductive tissues.

Clinically, our findings imply that LYG1 deficiency may serve as a genetic predisposing factor for inflammation-induced epididymal damage and infertility. Modulating LYG1-dependent pathways, such as ECM-receptor interaction or immune cell metabolism, could offer novel therapeutic strategies to attenuate fibrosis and preserve sperm quality in patients with chronic epididymitis (20). Additionally, sperm-specific LYG1 expression may serve as a biomarker for inflammatory sperm dysfunction, especially in cases where traditional semen analysis (e.g., motility, count) yields inconclusive results.

Limitations of this study include the lack of *in vivo* gain-of-function experiments with recombinant LYG1, primarily due to technical challenges in local delivery to the epididymis. Future studies will address this by investigating recombinant LYG1 in epididymal epithelial cell cultures to confirm direct effects on inflammatory and fibrotic pathways. Furthermore, exploring the potential interplay between LYG1 and other Lysozyme G-like family members (e.g., LYG2) will provide a more comprehensive understanding of their roles in reproductive tract inflammation.

## 5 Conclusion

LYG1 regulates the balance between inflammatory response and tissue repair in the epididymis, and its deficiency exacerbates LPS-induced epididymal fibrosis and sperm dysfunction. These findings deepen our understanding of the pathogenesis of chronic epididymitis and identify LYG1-dependent pathways as promising therapeutic targets for preserving male fertility.

## Data Availability

The datasets presented in this study can be found in online repositories. The names of the repository/repositories and accession number(s) can be found in the article/**Supplementary Material**.

## References

[1] Fijak M Pilatz A Hedger MP Nicolas N Bhushan S Michel V . Infectious, inflammatory and ‘autoimmune’ male factor infertility: how do rodent models inform clinical practice? Hum Reprod Update. (2018) 24:416–41. doi: 10.1093/humupd/dmy009, PMID: 29648649 PMC6016649

[2] Michel V Pilatz A Hedger MP Meinhardt A . Epididymitis: revelations at the convergence of clinical and basic sciences. Asian J Androl. (2015) 17:756–63. doi: 10.4103/1008-682X.155770, PMID: 26112484 PMC4577585

[3] Da Silva AAS BarraChina F Avenatti MC Elizagaray ML Bastepe I Sasso-Cerri E . Proton-secreting cells as drivers of inflammation and sperm dysfunction in LPS-induced epididymitis. Funct (Oxf). (2025) 6:zqaf023. doi: 10.1093/function/zqaf023, PMID: 40455583 PMC12203219

[4] Sarman E Koca HB . Effect of grape seed extract on doxorubicin-induced testicular and epididymal damage in rats. Hum Exp Toxicol. (2025) 44:9603271251319787. doi: 10.1177/09603271251319787, PMID: 40086075

[5] Liu H Zhang Y Liu Z Wang P Mo X Fu W . LYG1 exerts antitumor function through promoting the activation, proliferation, and function of CD4^+^ T cells. Oncoimmunology. (2017) 6:e1292195. doi: 10.1080/2162402X.2017.1292195, PMID: 28507796 PMC5414861

[6] Liu H Yu Z Tang B Miao S Qin C Li Y . LYG1 deficiency attenuates the severity of acute graft-versus-host disease via skewing allogeneic T cells polarization towards treg cells. Front Immunol. (2021) 12:647894. doi: 10.3389/fimmu.2021.647894, PMID: 34262560 PMC8273552

[7] Ahn SH Halgren K Grzesiak G MacRenaris KW Sue A Xie H . Autoimmune regulator deficiency causes sterile epididymitis and impacts male fertility through disruption of inorganic physiology. J Immunol. (2025) 214:1504–16. doi: 10.1093/jimmun/vkaf054, PMID: 40267393 PMC12311386

[8] Wijayarathna R Pasalic A Nicolas N Biniwale S Ravinthiran R Genovese R . Region-specific immune responses to autoimmune epididymitis in the murine reproductive tract. Cell Tissue Res. (2020) 381:351–60. doi: 10.1007/s00441-020-03215-8, PMID: 32383098

[9] Liu JC Wang P Zeng QX Yang C Lyu M Li Y . Myd88 signaling is involved in the inflammatory response in LPS-induced mouse epididymitis and bone-marrow-derived dendritic cells. Int J Mol Sci. (2023) 24:7838. doi: 10.3390/ijms24097838, PMID: 37175545 PMC10178089

[10] Zi T Liu Y Wang Z Zhang Y Xie M Zhu P . Melatonin alleviates oxidative damage in mouse spermatogenesis and sperm quality parameters induced by exposure to bisphenol A. Ecotoxicoloby Environ Safety. (2023) 253:114709. doi: 10.1016/j.ecoenv.2023.114709

[11] Liu F Liu X Liu X Li T Zhu P Liu Z . Integrated analyses of phenotype and quantitative proteome of CMTM4 deficient mice reveal its association with male fertility. Mol Cell Proteomics. (2019) 18:1070–84. doi: 10.1074/mcp.RA119.001416, PMID: 30867229 PMC6553932

[12] Zhang Y Liu Y Teng Z Wang Z Zhu P Wang Z . Human umbilical cord mesenchymal stem cells (hUC-MSCs) alleviate paclitaxel-induced spermatogenesis defects and maintain male fertility. Biol Res. (2023) 56:47. doi: 10.1186/s40659-023-00459-w, PMID: 37574561 PMC10424423

[13] Khan R Azhar M Umair M . Decoding the genes orchestrating egg and sperm fusion reactions and their roles in fertility. Biomedicines. (2024) 12:2850. doi: 10.3390/biomedicines12122850, PMID: 39767756 PMC11673484

[14] Nayyab S Gervasi MG Tourzani DA Shamailova Y Akizawa H Taghavi M . Identification of TSSK1 and TSSK2 as novel targets for male contraception. Biomolecules. (2025) 15:601. doi: 10.3390/biom15040601, PMID: 40305308 PMC12024862

[15] Liu X Wang X Liu F . Decreased expression of heat shock protein A4L in spermatozoa is positively related to poor human sperm quality. Mol Reprod Dev. (2019) 86:379–86. doi: 10.1002/mrd.23113, PMID: 30637842

[16] Zhou X Liu Z Jia W Hou M Zhang X . Actl7a deficiency in mice leads to male infertility and fertilization failure. Biochem Biophys Res Commun. (2022) 623:154–61. doi: 10.1016/j.bbrc.2022.07.065, PMID: 35921706

[17] Cheng Y Shao Y . Identification of novel diagnostic markers for atherosclerosis using machine-learning algorithms. J Coll Physicians Surg Pak. (2025) 35:574–9. doi: 10.29271/jcpsp.2025.05.574, PMID: 40325572

[18] Naganuma T Takagi S Kanetake T Kitamura T Hattori S Miyakawa T . Disruption of the sjögren-larsson syndrome gene aldh3a2 in mice increases keratinocyte growth and retards skin barrier recovery. J Biol Chem. (2016) 291:11676–88. doi: 10.1074/jbc.M116.714030, PMID: 27053112 PMC4882436

[19] Sayed MAM Hussein MT Mustafa FEA Abdelhefeez E Hussein AMA Abdelfattah MG . Attenuation of chronic oxidative stress-induced testicular and epididymal dysfunction by oral intake of lepidium meyenii in New Zealand rabbits. J Anim Physiol Anim Nutr (Berl). (2025) 109:682–700. doi: 10.1111/jpn.14083, PMID: 39710993

[20] Gao S Chen Z Shi J Chen Z Yun D Li X . Sperm immotility is associated with epididymis metabolism disorder in mice under obstructive azoospermia. FASEB J. (2023) 37:e23081. doi: 10.1096/fj.202201862RR, PMID: 37410071

