# Supplementary figures and images for: Lyg1 deficiency aggravated LPS-induced chronic epididymal inflammation and sperm dysfunction in mouse
Source: Front Immunol. 2025 Dec 9;16:1699581. doi: 10.3389/fimmu.2025.1699581 (PMC12722883; doi:10.3389/fimmu.2025.1699581)

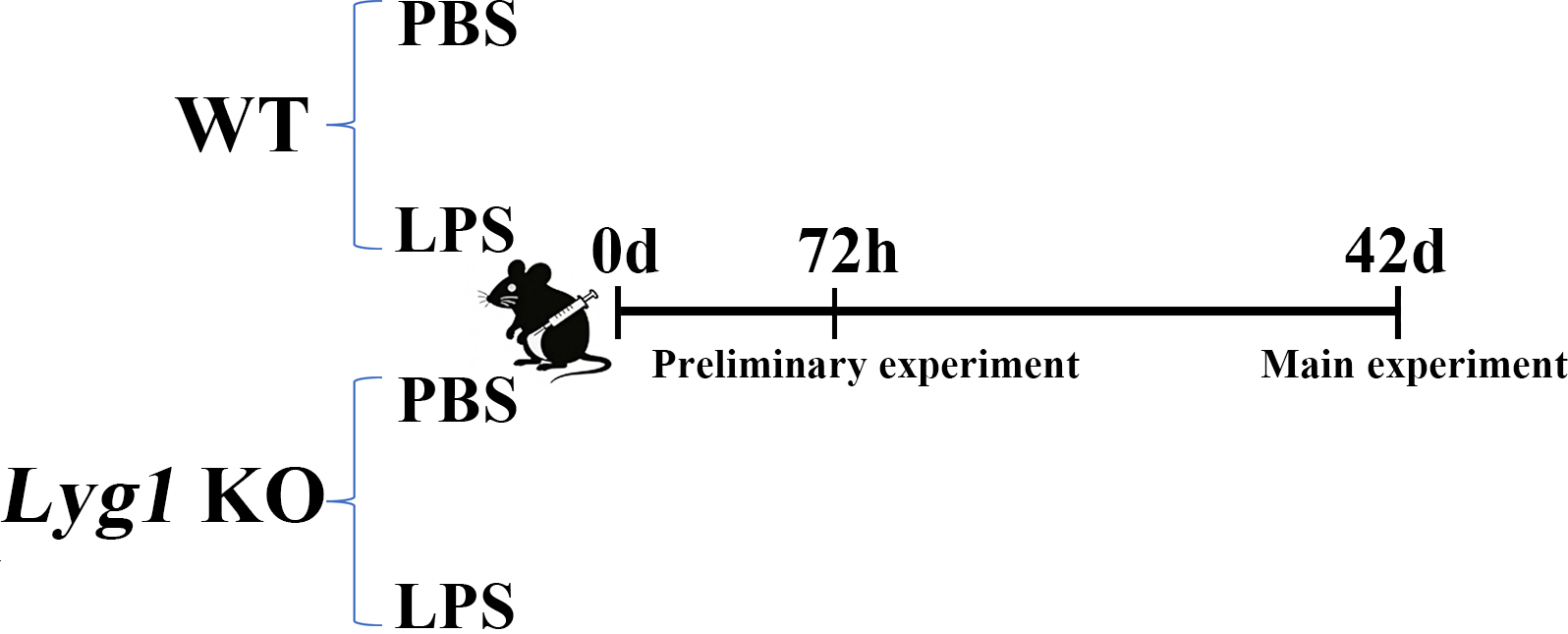

Supplement: Supplementary file 1 [file Image1.jpeg]

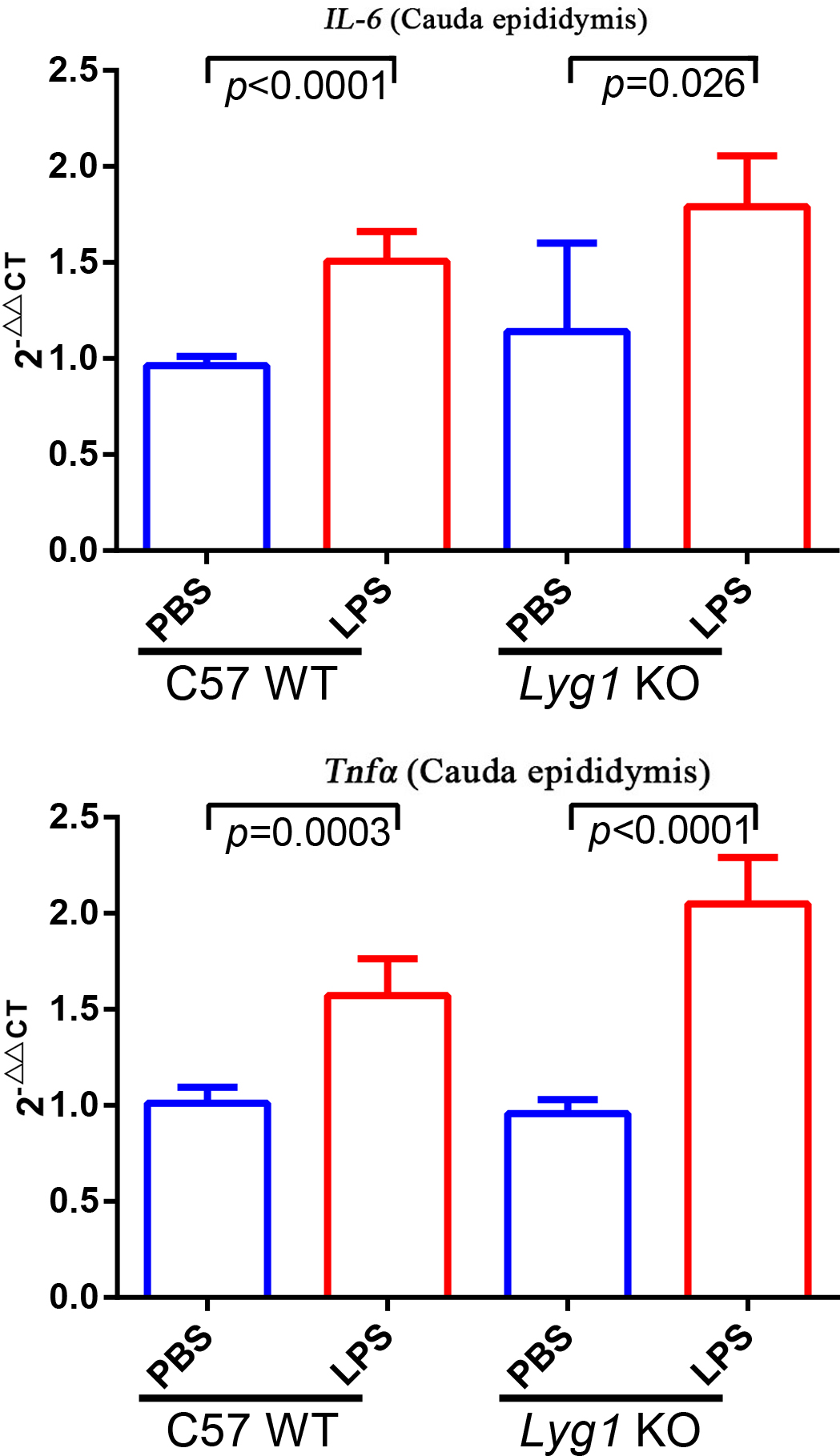

Supplement: Supplementary file 2 [file Image2.jpeg]

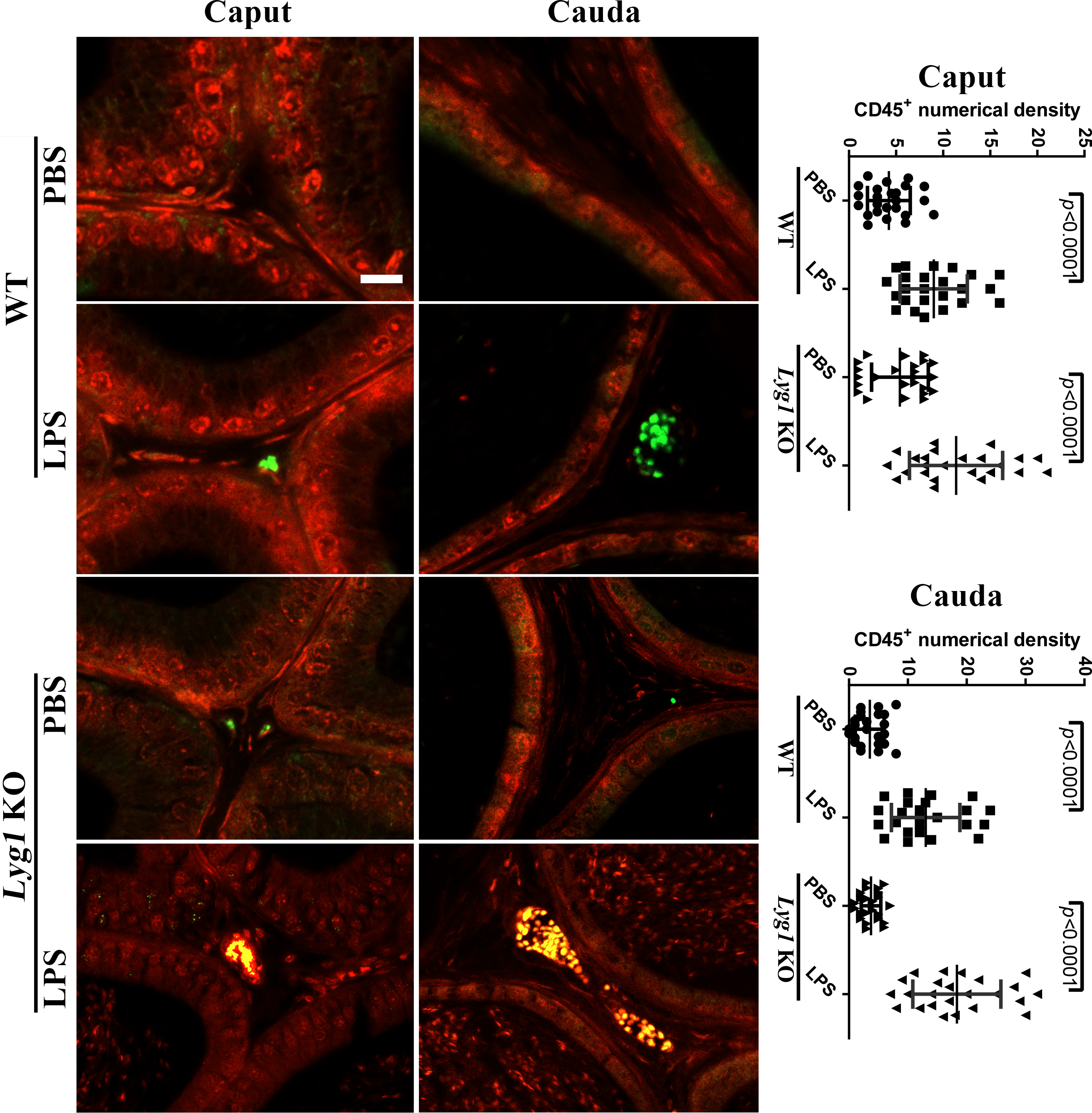

Supplement: Supplementary file 3 [file Image3.jpeg]

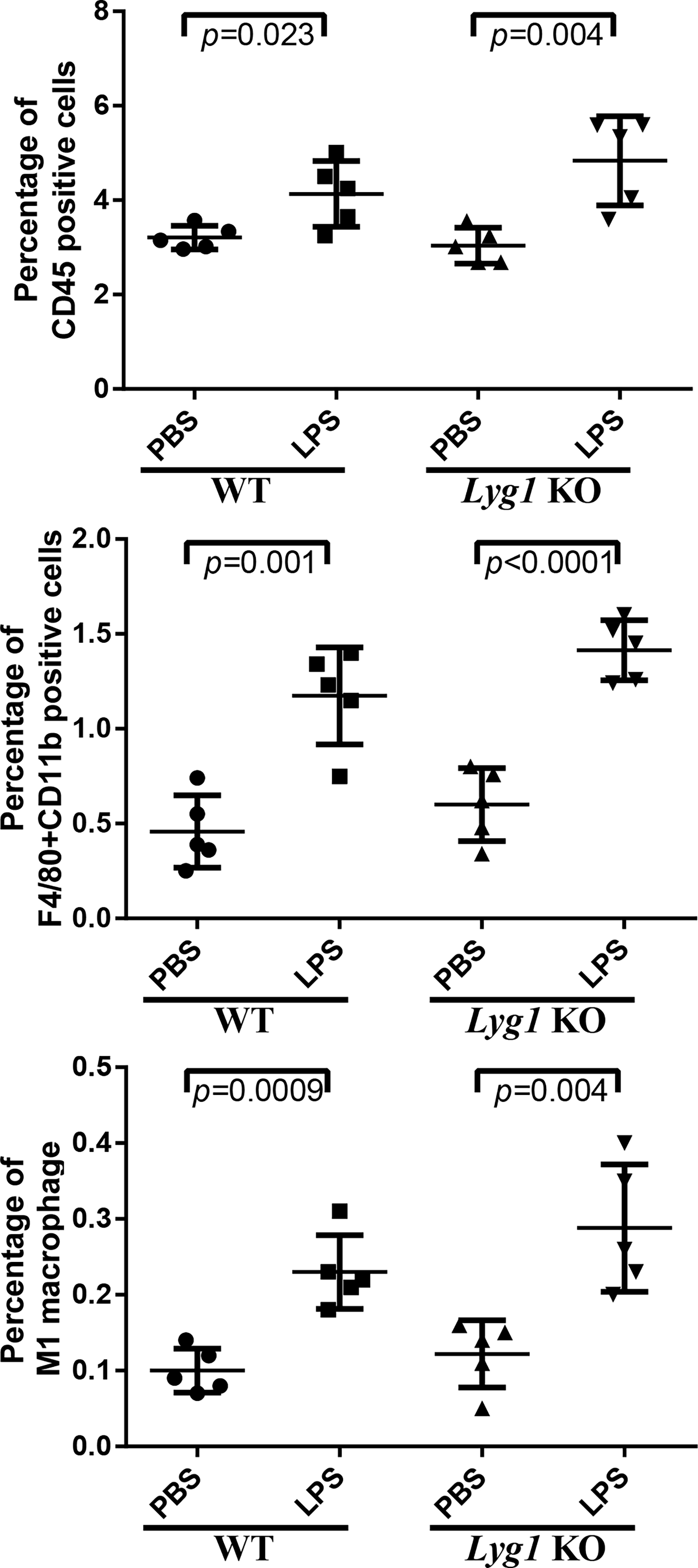

Supplement: Supplementary file 4 [file Image4.jpeg]

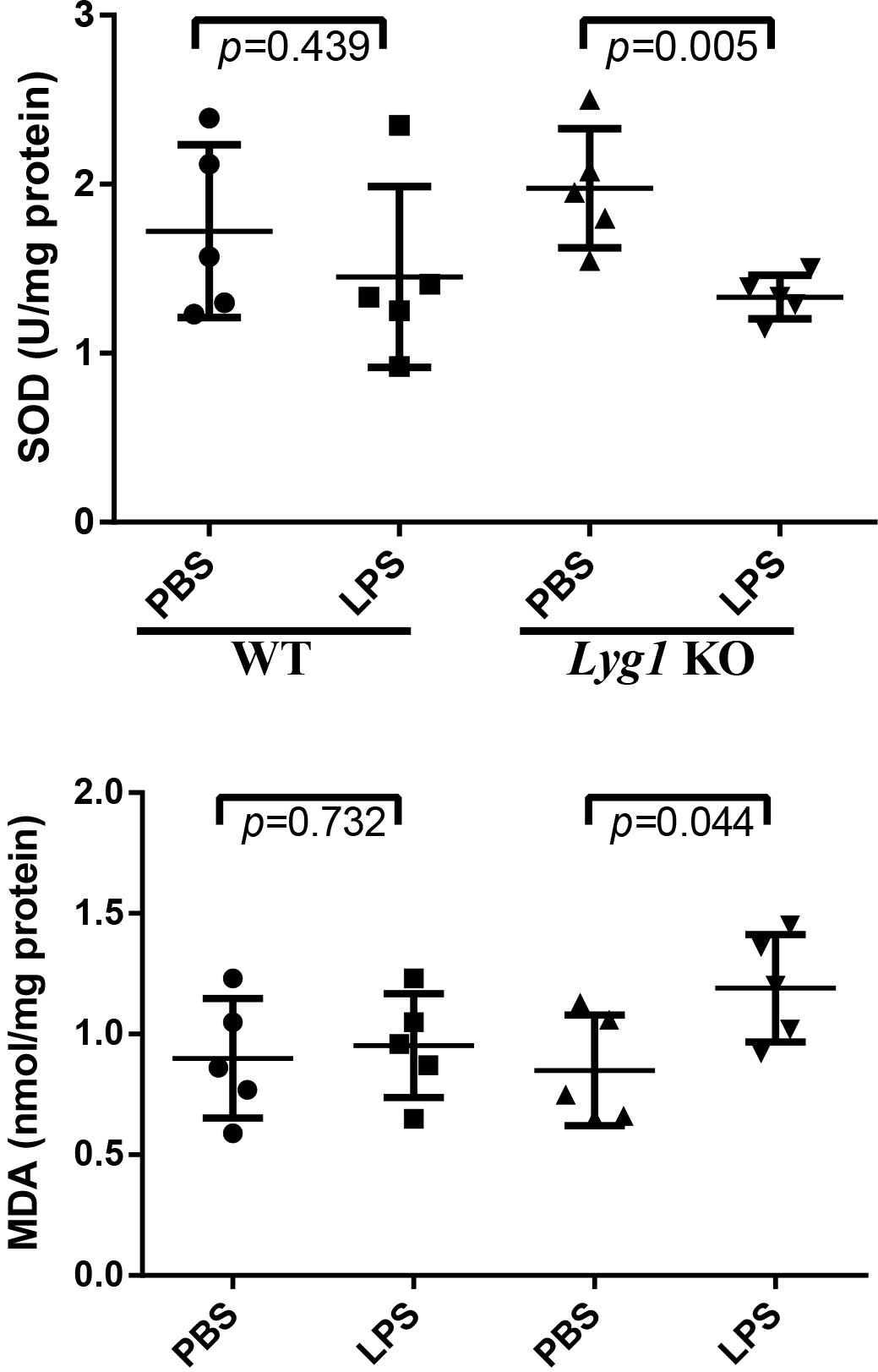

Supplement: Supplementary file 5 [file Image5.jpeg]

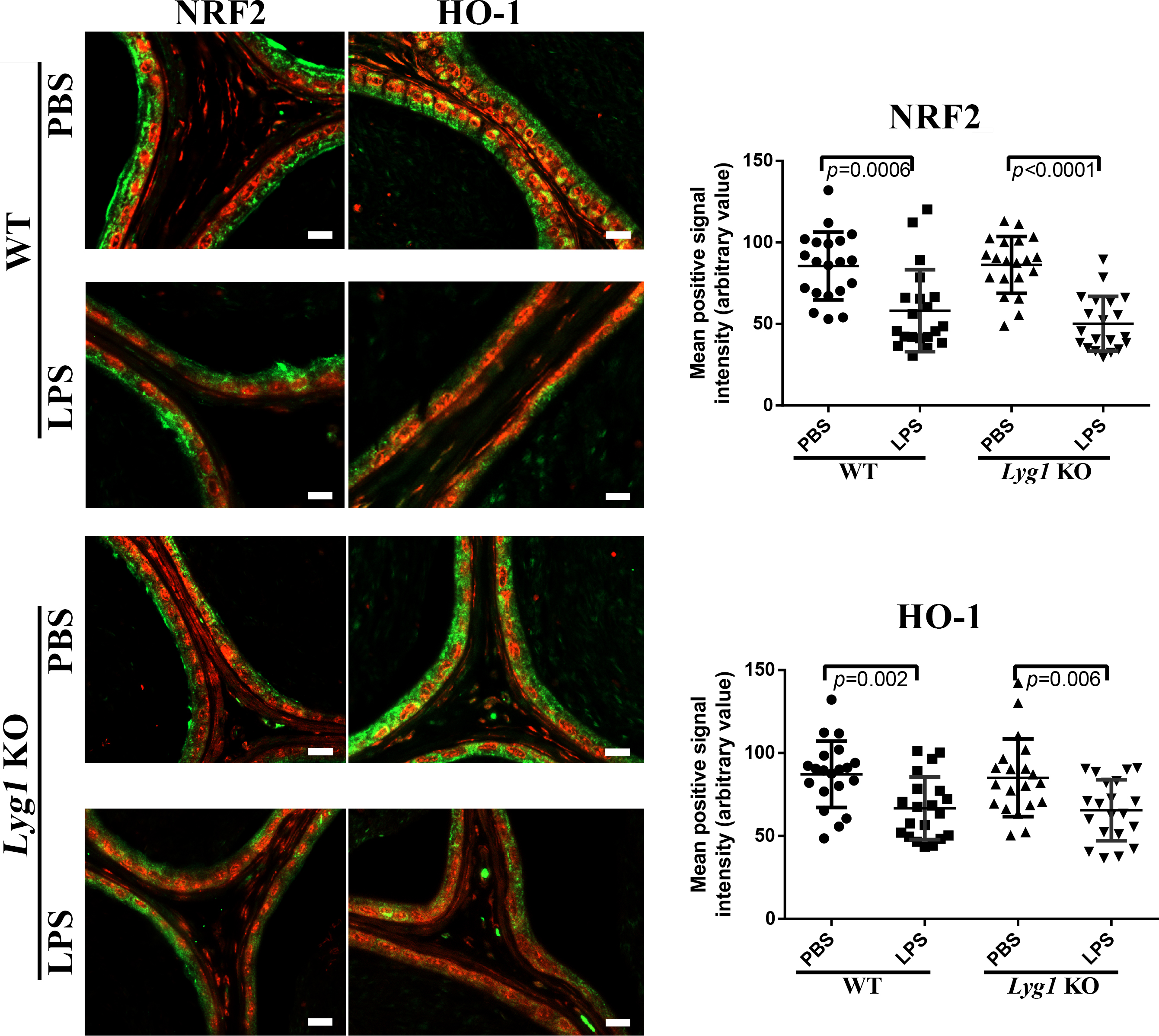

Supplement: Supplementary file 6 [file Image6.jpeg]

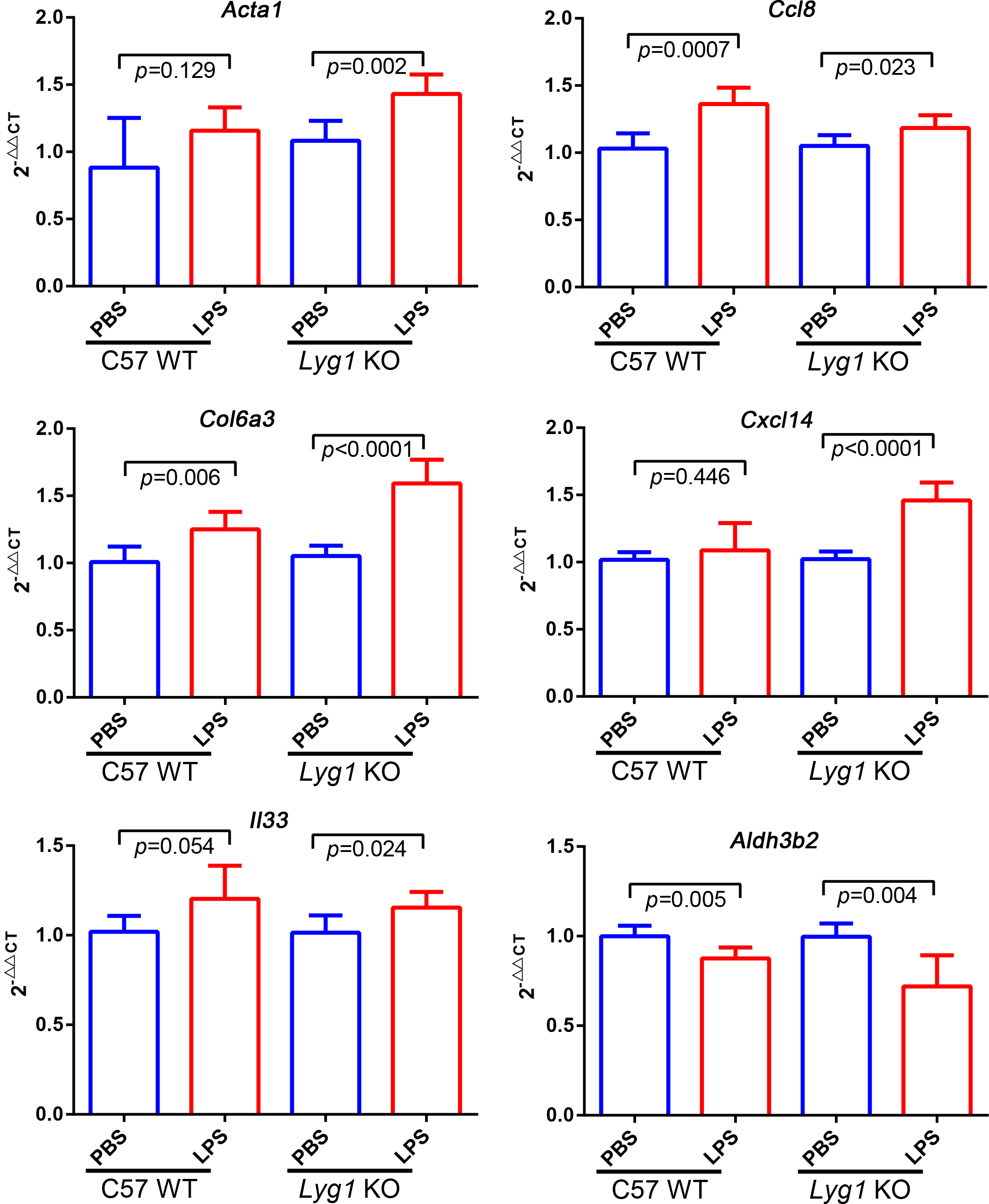

Supplement: Supplementary file 7 [file Image7.jpeg]
