# Supplementary material for: Lyg1 deficiency aggravated LPS-induced chronic epididymal inflammation and sperm dysfunction in mouse
Source: Front Immunol. 2025 Dec 9;16:1699581. doi: 10.3389/fimmu.2025.1699581 (PMC12722883; doi:10.3389/fimmu.2025.1699581)
Supplement: Supplementary file 8 [file Table1.docx]

**Supplementary Table 1. List of differentially expressed genes in LPS-induced epididymitis (WT mice)**

| **Up-regulated genes in LPS-treated mice epididymis** | | |
| --- | --- | --- |
| **gene_name** | **Fold** | ***p* value** |
| **Smim24** | **4.83** | **0.049** |
| **Retnla** | **4.50** | **0.020** |
| **Gapdhs** | **4.31** | **0.041** |
| **Wbscr25** | **4.22** | **0.023** |
| **Pi16** | **3.88** | **0.048** |
| **Ctla2a** | **3.85** | **0.030** |
| **Il31ra** | **3.73** | **0.041** |
| **Ccl8** | **2.99** | **0.044** |
| **Spa17** | **2.90** | **0.039** |
| **Clec3b** | **2.71** | **0.011** |
| **Lpin1** | **2.69** | **0.016** |
| **Sord** | **2.69** | **0.043** |
| **Akap12** | **2.58** | **0.022** |
| **Ccdc136** | **2.51** | **0.039** |
| **Lrrc57** | **2.47** | **0.019** |
| **Hk1** | **2.40** | **0.019** |
| **1700001O22Rik** | **2.27** | **0.048** |
| **Clpb** | **2.20** | **0.033** |
| **Slit3** | **2.19** | **0.048** |
| **Osgin1** | **2.06** | **0.048** |
| **Lbp** | **2.03** | **0.019** |
| **St6galnac2** | **1.99** | **0.007** |
| **Scara5** | **1.94** | **0.019** |
| **Wnt7b** | **1.93** | **0.004** |
| **Dcun1d1** | **1.93** | **0.048** |
| **Spns3** | **1.91** | **0.050** |
| **Dcn** | **1.91** | **0.050** |
| **Odf2** | **1.90** | **0.046** |
| **Rgs5** | **1.89** | **0.023** |
| **Aqp11** | **1.88** | **0.016** |
| **Cygb** | **1.88** | **0.046** |
| **Plscr2** | **1.85** | **0.044** |
| **Sorbs1** | **1.80** | **0.050** |
| **Il33** | **1.80** | **0.045** |
| **Mapk6** | **1.80** | **0.010** |
| **Folr2** | **1.79** | **0.020** |
| **Rnf138** | **1.78** | **0.040** |
| **Nupr1l** | **1.77** | **0.046** |
| **Ube2n** | **1.76** | **0.028** |
| **Defb26** | **1.75** | **0.048** |
| **Lasp1** | **1.74** | **0.028** |
| **Lipe** | **1.71** | **0.004** |
| **Utrn** | **1.71** | **0.022** |
| **Gsn** | **1.71** | **0.049** |
| **Ogn** | **1.70** | **0.031** |
| **Gab2** | **1.70** | **0.014** |
| **Plekho2** | **1.68** | **0.011** |
| **Fam46c** | **1.68** | **0.012** |
| **Heg1** | **1.67** | **0.018** |
| **Serping1** | **1.67** | **0.039** |
| **Mea1** | **1.65** | **0.032** |
| **Fstl1** | **1.65** | **0.044** |
| **Slit1** | **1.65** | **0.011** |
| **Irs2** | **1.64** | **0.019** |
| **Timp2** | **1.64** | **0.042** |
| **Vim** | **1.63** | **0.024** |
| **Arhgdib** | **1.61** | **0.043** |
| **Mustn1** | **1.61** | **0.020** |
| **Hivep1** | **1.61** | **0.003** |
| **Lypd6** | **1.60** | **0.033** |
| **Rnaseh2a** | **1.60** | **0.015** |
| **Sptb** | **1.58** | **0.033** |
| **Tgfbr2** | **1.58** | **0.036** |
| **Igfbp3** | **1.58** | **0.019** |
| **Sparc** | **1.57** | **0.042** |
| **Dzip1** | **1.55** | **0.023** |
| **Dyrk1b** | **1.55** | **0.057** |
| **5031439G07Rik** | **1.54** | **0.016** |
| **Phf7** | **1.54** | **0.044** |
| **Cyb5d1** | **1.53** | **0.015** |
| **1700001K19Rik** | **1.53** | **0.007** |
| **Gcnt4** | **1.53** | **0.000** |
| **Rab31** | **1.52** | **0.045** |
| **Nid1** | **1.52** | **0.044** |
| **Rps6** | **1.52** | **0.014** |
| **Wwtr1** | **1.52** | **0.033** |
| **Rara** | **1.51** | **0.014** |
| **Mturn** | **1.51** | **0.013** |
| **Itih5** | **1.51** | **0.056** |
| **Csgalnact2** | **1.50** | **0.010** |
| **Dcbld2** | **1.50** | **0.016** |
| **Down-regulated genes in LPS-treated mice epididymis** | | |
| **gene_name** | **Fold** | **p value** |
| **Rnf5** | **0.66** | **0.011** |
| **9330020H09Rik** | **0.65** | **0.019** |
| **Lor** | **0.65** | **0.014** |
| **Amigo3** | **0.65** | **0.001** |
| **Rnf8** | **0.65** | **0.007** |
| **Mmp28** | **0.64** | **0.025** |
| **March9** | **0.64** | **0.007** |
| **Cstad** | **0.64** | **0.044** |
| **Aldh3b2** | **0.63** | **0.047** |
| **Vipr1** | **0.63** | **0.032** |
| **S100a14** | **0.63** | **0.005** |
| **Rnase6** | **0.62** | **0.041** |
| **Tnfrsf25** | **0.62** | **0.047** |
| **Eif4ebp3** | **0.62** | **0.039** |
| **Hoxb3** | **0.62** | **0.002** |
| **A930002H24Rik** | **0.61** | **0.012** |
| **Efcab12** | **0.61** | **0.002** |
| **4931428F04Rik** | **0.61** | **0.004** |
| **B130055M24Rik** | **0.61** | **0.047** |
| **Mir5119** | **0.60** | **0.050** |
| **Zscan30** | **0.60** | **0.020** |
| **Mir6999** | **0.59** | **0.041** |
| **Gadd45a** | **0.59** | **0.038** |
| **Col7a1** | **0.58** | **0.040** |
| **Sox9** | **0.58** | **0.043** |
| **Neurl2** | **0.58** | **0.043** |
| **Rac3** | **0.58** | **0.016** |
| **Calml3** | **0.57** | **0.028** |
| **Plau** | **0.57** | **0.009** |
| **Lrrtm1** | **0.56** | **0.020** |
| **Adgrf3** | **0.55** | **0.009** |
| **Lsm7** | **0.55** | **0.031** |
| **Ighj2** | **0.55** | **0.041** |
| **Klk1b5** | **0.54** | **0.009** |
| **Mir466i** | **0.53** | **0.019** |
| **Mir345** | **0.51** | **0.042** |
| **Krt15** | **0.51** | **0.051** |
| **Snord58b** | **0.51** | **0.022** |
| **Atp4a** | **0.49** | **0.036** |
| **Lgals7** | **0.48** | **0.041** |
| **Apoa2** | **0.47** | **0.041** |
| **Mir6948** | **0.38** | **0.039** |
| **Alb** | **0.34** | **0.028** |
| **Snord66** | **0.29** | **0.042** |
| **Mir5100** | **0.15** | **0.030** |
