# Supplementary material for: Lyg1 deficiency aggravated LPS-induced chronic epididymal inflammation and sperm dysfunction in mouse
Source: Front Immunol. 2025 Dec 9;16:1699581. doi: 10.3389/fimmu.2025.1699581 (PMC12722883; doi:10.3389/fimmu.2025.1699581)
Supplement: Supplementary file 9 [file Table2.docx]

**Supplementary Table 2. List of differentially expressed genes in LPS-induced epididymitis (Lyg1 KO mice)**

| **Up-regulated genes in LPS-treated mice epididymis** | | |
| --- | --- | --- |
| **gene_name** | **Fold** | **p value** |
| **Slc15a2** | **8.03** | **0.037** |
| **Acsbg1** | **3.13** | **0.013** |
| **Lce6a** | **3.07** | **0.047** |
| **Mir26b** | **2.90** | **0.027** |
| **Acta1** | **2.89** | **0.048** |
| **Cxcl14** | **2.64** | **0.041** |
| **Treh** | **2.55** | **0.039** |
| **Gimap4** | **2.51** | **0.035** |
| **Thbs1** | **2.46** | **0.007** |
| **6330403K07Rik** | **2.43** | **0.034** |
| **Lsp1** | **2.37** | **0.015** |
| **Csf1r** | **2.37** | **0.017** |
| **Ly86** | **2.36** | **0.049** |
| **Mmp2** | **2.35** | **0.049** |
| **Pi16** | **2.34** | **0.032** |
| **9230112D13Rik** | **2.33** | **0.049** |
| **Defb20** | **2.27** | **0.042** |
| **Oaf** | **2.24** | **0.046** |
| **Fam46b** | **2.23** | **0.011** |
| **Coro1a** | **2.22** | **0.046** |
| **Sfrp2** | **2.22** | **0.049** |
| **Ccdc102a** | **2.22** | **0.045** |
| **Hoxc10** | **2.17** | **0.041** |
| **Card9** | **2.14** | **0.049** |
| **Tnc** | **2.12** | **0.046** |
| **Ogn** | **2.07** | **0.044** |
| **Serpinf1** | **2.06** | **0.038** |
| **Itga8** | **2.05** | **0.043** |
| **Nptx2** | **2.03** | **0.047** |
| **Dnase1l2** | **2.03** | **0.044** |
| **Tcf21** | **2.02** | **0.039** |
| **Thy1** | **1.97** | **0.047** |
| **Fxyd3** | **1.97** | **0.040** |
| **Susd2** | **1.96** | **0.041** |
| **Faim2** | **1.95** | **0.004** |
| **Hoxa11** | **1.94** | **0.024** |
| **Popdc2** | **1.94** | **0.041** |
| **Tnfsf13b** | **1.93** | **0.030** |
| **Tnfrsf14** | **1.93** | **0.046** |
| **Igfbp6** | **1.91** | **0.049** |
| **Col6a3** | **1.91** | **0.042** |
| **Gpsm3** | **1.90** | **0.042** |
| **Mus81** | **1.89** | **0.048** |
| **Nkd2** | **1.89** | **0.048** |
| **Ctgf** | **1.88** | **0.045** |
| **Fgd2** | **1.87** | **0.045** |
| **Olfml3** | **1.86** | **0.060** |
| **Ndufaf5** | **1.86** | **0.046** |
| **Neurl2** | **1.86** | **0.003** |
| **Fermt3** | **1.86** | **0.042** |
| **Sdc2** | **1.85** | **0.044** |
| **Spns3** | **1.84** | **0.050** |
| **Tspan18** | **1.84** | **0.043** |
| **Snord104** | **1.83** | **0.045** |
| **Cnn2** | **1.82** | **0.043** |
| **Cygb** | **1.81** | **0.051** |
| **Smim1** | **1.81** | **0.062** |
| **Pth1r** | **1.79** | **0.043** |
| **Fcer1g** | **1.79** | **0.046** |
| **Emp3** | **1.78** | **0.046** |
| **P4ha1** | **1.76** | **0.017** |
| **Bgn** | **1.74** | **0.048** |
| **Timp2** | **1.71** | **0.044** |
| **Rbpms2** | **1.70** | **0.047** |
| **Dao** | **1.70** | **0.011** |
| **Rnd3** | **1.70** | **0.000** |
| **Col18a1** | **1.70** | **0.036** |
| **Fmod** | **1.69** | **0.046** |
| **Lmod1** | **1.68** | **0.041** |
| **Lims2** | **1.68** | **0.036** |
| **Prrx1** | **1.68** | **0.058** |
| **Tpm1** | **1.68** | **0.044** |
| **Fam189b** | **1.66** | **0.041** |
| **Sdc3** | **1.66** | **0.040** |
| **Dok4** | **1.65** | **0.027** |
| **Arrb2** | **1.65** | **0.044** |
| **Tinagl1** | **1.65** | **0.034** |
| **1500011K16Rik** | **1.65** | **0.044** |
| **Gpc6** | **1.64** | **0.045** |
| **Tspan2** | **1.64** | **0.040** |
| **Rab8b** | **1.64** | **0.053** |
| **Ldlr** | **1.63** | **0.055** |
| **Mmp11** | **1.63** | **0.080** |
| **Icosl** | **1.63** | **0.050** |
| **Aebp1** | **1.63** | **0.053** |
| **Hmha1** | **1.63** | **0.008** |
| **Gjc1** | **1.63** | **0.041** |
| **Dmpk** | **1.62** | **0.049** |
| **Arhgap10** | **1.61** | **0.042** |
| **Mxra8** | **1.61** | **0.042** |
| **Trpv2** | **1.61** | **0.043** |
| **Prrg3** | **1.61** | **0.050** |
| **Snrpg** | **1.60** | **0.007** |
| **0610009L18Rik** | **1.59** | **0.009** |
| **Sult1a1** | **1.59** | **0.045** |
| **Gja4** | **1.59** | **0.049** |
| **Pld4** | **1.59** | **0.010** |
| **Jam3** | **1.59** | **0.046** |
| **Rgs19** | **1.58** | **0.035** |
| **Snhg18** | **1.58** | **0.048** |
| **Adamtsl5** | **1.58** | **0.049** |
| **Trip6** | **1.57** | **0.042** |
| **Slc24a3** | **1.57** | **0.047** |
| **AA986860** | **1.57** | **0.044** |
| **Zfp385a** | **1.57** | **0.004** |
| **Nexn** | **1.57** | **0.043** |
| **Serpinh1** | **1.56** | **0.046** |
| **Sirpa** | **1.56** | **0.011** |
| **Itga7** | **1.56** | **0.050** |
| **Lmcd1** | **1.56** | **0.041** |
| **Cemip** | **1.56** | **0.050** |
| **Hspb2** | **1.55** | **0.030** |
| **Laptm5** | **1.54** | **0.045** |
| **Pmepa1** | **1.54** | **0.049** |
| **Mrps36** | **1.53** | **0.048** |
| **Adrb2** | **1.53** | **0.046** |
| **Sept1** | **1.52** | **0.046** |
| **Acvrl1** | **1.52** | **0.045** |
| **Amotl2** | **1.52** | **0.048** |
| **Dennd1c** | **1.52** | **0.049** |
| **Slc9a3r2** | **1.51** | **0.040** |
| **Smad6** | **1.51** | **0.046** |
| **P3h3** | **1.51** | **0.047** |
| **Ecm1** | **1.51** | **0.050** |
| **Clmp** | **1.51** | **0.050** |
| **Cd59b** | **1.50** | **0.048** |
| **Arhgef25** | **1.50** | **0.046** |
| **Glipr2** | **1.50** | **0.048** |
| **Down-regulated genes in LPS-treated mice epididymis** | | |
| **gene_name** | **Fold** | **p value** |
| **Ubxn2a** | **0.67** | **0.028** |
| **Ddx19b** | **0.66** | **0.041** |
| **Ptpn3** | **0.66** | **0.034** |
| **Ptpru** | **0.66** | **0.046** |
| **Cgnl1** | **0.66** | **0.049** |
| **Stc1** | **0.65** | **0.067** |
| **E330009J07Rik** | **0.65** | **0.017** |
| **Tfcp2l1** | **0.65** | **0.038** |
| **Lad1** | **0.65** | **0.033** |
| **Pitpnc1** | **0.65** | **0.046** |
| **Eif4ebp2** | **0.65** | **0.041** |
| **Defb39** | **0.64** | **0.035** |
| **Camsap3** | **0.64** | **0.050** |
| **Ces1e** | **0.63** | **0.064** |
| **Hpn** | **0.63** | **0.044** |
| **Sptbn2** | **0.62** | **0.042** |
| **9230110C19Rik** | **0.62** | **0.049** |
| **Azgp1** | **0.62** | **0.042** |
| **Notch3** | **0.62** | **0.048** |
| **Fzd4** | **0.62** | **0.043** |
| **Abca1** | **0.62** | **0.050** |
| **Gga2** | **0.61** | **0.008** |
| **Simc1** | **0.61** | **0.014** |
| **n-R5s29** | **0.60** | **0.019** |
| **Muc20** | **0.59** | **0.026** |
| **C77080** | **0.59** | **0.053** |
| **Ace** | **0.59** | **0.043** |
| **Lonrf1** | **0.59** | **0.051** |
| **Defb37** | **0.58** | **0.011** |
| **Rragd** | **0.58** | **0.050** |
| **Cpsf4l** | **0.57** | **0.052** |
| **Wee1** | **0.57** | **0.048** |
| **Syn2** | **0.57** | **0.050** |
| **Bsnd** | **0.56** | **0.020** |
| **Mertk** | **0.56** | **0.051** |
| **Pstpip2** | **0.56** | **0.025** |
| **Fam149a** | **0.55** | **0.030** |
| **Aldh3b2** | **0.54** | **0.049** |
| **Tm4sf4** | **0.54** | **0.012** |
| **Prkar2b** | **0.53** | **0.067** |
| **Abhd3** | **0.51** | **0.006** |
| **Lrrtm1** | **0.49** | **0.048** |
| **Slc6a20a** | **0.49** | **0.007** |
| **Pxmp2** | **0.45** | **0.004** |
| **Etv5** | **0.45** | **0.046** |
| **Capn8** | **0.43** | **0.045** |
| **Odam** | **0.40** | **0.033** |
| **Rn7sk** | **0.40** | **0.000** |
| **Crisp4** | **0.40** | **0.017** |
| **Mir8094** | **0.36** | **0.007** |
| **Arnt2** | **0.36** | **0.042** |
| **Mir6386** | **0.36** | **0.017** |
| **Snord17** | **0.35** | **0.052** |
| **Malrd1** | **0.35** | **0.006** |
| **Dkkl1** | **0.33** | **0.011** |
| **Mir26a-1** | **0.28** | **0.020** |
| **Xkrx** | **0.28** | **0.044** |
| **Ace2** | **0.26** | **0.035** |
| **Pate2** | **0.23** | **0.006** |
| **Defb18** | **0.21** | **0.033** |
| **Rnase1** | **0.15** | **0.048** |
