# Supplementary material for: Lyg1 deficiency aggravated LPS-induced chronic epididymal inflammation and sperm dysfunction in mouse
Source: Front Immunol. 2025 Dec 9;16:1699581. doi: 10.3389/fimmu.2025.1699581 (PMC12722883; doi:10.3389/fimmu.2025.1699581)
Supplement: Supplementary file 10 [file Table3.docx]

Supplementary Table 3. List of primers for representative genes

| **Genes** |  | **Primers** |
| --- | --- | --- |
| *Actb* | Forward | GCAGCTCAGTAACAGTCCGC |
|  | Reverse | AGTGTGACGTTGACATCCGT |
| *Lyg1* | Forward | CTTCTGAGACATCAACCCACC |
|  | Reverse | CAAGTCCACTGCCAATGTTG |
| *Lyg2* | Forward | CCCAAAGGATAAGAGTCACGG |
|  | Reverse | CATGGTACAGACGAGGGTG |
| *Tnfa* | Forward | CATCTTCTCAAAATTCGAGTGACAA |
|  | Reverse | TGGGAGTAGACAAGGTACAACCC |
| *Il6* | Forward | GAGGATACCACTCCCAACAGACC |
|  | Reverse | AAGTGCATCATCGTTGTTCATACA |
| *Acta1* | Forward | CTCCCTGGAGAAGAGCTATGA |
|  | Reverse | CGATAAAGGAAGGCTGGAAGAG |
| *Ccl8* | Forward | AAGATCTACGCAGTGCTTCTTT |
|  | Reverse | AGGTGACTGGAGCCTTATCT |
| *Col6a3* | Forward | GAGAGCTGCCCAACATAGAA |
|  | Reverse | CCGGGAAGGAGAAGGTAAATC |
| *Cxcl14* | Forward | CTGCGAGGAGAAGATGGTTATC |
|  | Reverse | CTTCTCGTTCCAGGCATTGTA |
| *Il33* | Forward | TCCACGGGATTCTAGGAAGA |
|  | Reverse | GAGGCAGGAGACTGTGTTAAA |
| *Aldh3b2* | Forward | CAGGTACCATGGCAAGTTCT |
|  | Reverse | CGGGTAACGGAGGTCATTAAG |
