# Supplementary material for: Lyg1 deficiency aggravated LPS-induced chronic epididymal inflammation and sperm dysfunction in mouse
Source: Front Immunol. 2025 Dec 9;16:1699581. doi: 10.3389/fimmu.2025.1699581 (PMC12722883; doi:10.3389/fimmu.2025.1699581)
Supplement: Supplementary file 11 [file DataSheet1.pdf]

Supplementary Figure Legends

Supplementary Figure 1

Schematic diagram of the experimental design. A total of 64 mice were randomly divided into four groups: WT+PBS, WT+LPS, Lyg1 KO+PBS, and Lyg1 KO+LPS. For the preliminary experiment, 4 mice per group were sacrificed 72 hours post-treatment to detect cytokine expression (IL-6 and TNF-α) for model validation. For the main experiment, 12 mice per group were maintained until day 42 post-treatment to analyze epididymal histology, sperm function, oxidative stress, transcriptomic profiles, and in vitro fertilization outcomes.

Supplementary Figure 2

Validation of the LPS-induced chronic epididymitis model by cytokine expression. Relative mRNA expression levels of pro-inflammatory cytokines IL-6 (A) and TNF-α (B) in the caput and cauda epididymis of WT and Lyg1 KO mice 72 hours after PBS or LPS injection. Data are presented as mean ± SD (n=4 per group). Statistical analysis was performed by One-Way ANOVA followed by Tukey's post-hoc test. p value less than 0.05 was considered significance

Supplementary Figure 3

Quantification of $CD45^+$ leukocyte infiltration in the epididymis. Immunofluorescence staining of $CD45^+$ cells in the caput (A) and cauda (B) epididymis of WT and Lyg1 KO mice treated with PBS or LPS. The numerical density (Nv) of $CD45^+$ cells was calculated as cells/mm². Data are presented as mean ± SD (n=3 per group, 8 non-overlapping fields per mouse). Statistical analysis was performed by One-Way ANOVA followed by Tukey's post-hoc test. p value less than 0.05 was considered significance

Supplementary Figure 4

Flow cytometric analysis of immune cell subsets in the cauda epididymis. Proportions of $CD45^+$ leukocytes (A), $F4/80^+$ immune cells (B), and M1 macrophages (C) in the cauda epididymis of WT and Lyg1 KO mice treated with PBS or LPS. Data are presented as mean ± SD (n=3 per group). Statistical analysis was performed by One-Way ANOVA followed by Tukey's post-hoc test. p value less than 0.05 was considered significance

Supplementary Figure 5

Detection of oxidative stress indicators in the cauda epididymis. (A) Malondialdehyde (MDA) content (nmol/mg protein) and (B) superoxide dismutase (SOD) activity (U/mg protein) in cauda epididymis homogenates of WT and Lyg1 KO mice treated with PBS or LPS. Data are presented as mean ± SD (n=3 per group). Statistical analysis

was performed by One-Way ANOVA followed by Tukey's post-hoc test. p value less than 0.05 was considered significance

Supplementary Figure 6

Immunofluorescence analysis of NRF2 and HO-1 expression in the cauda epididymis. Relative fluorescence intensity of NRF2 (A) and HO-1 (B) in the cauda epididymis of WT and Lyg1 KO mice treated with PBS or LPS. Data are presented as mean ± SD (n=3 per group, ≥20 lumens analyzed per mouse). Statistical analysis was performed by One-Way ANOVA followed by Tukey's post-hoc test. p value less than 0.05 was considered significance

Supplementary Figure 7.

Bioinformatics analysis of differentially expressed genes (DEGs) in mouse cauda epididymis treated with PBS versus LPS. The bioinformatics analysis was conducted using the DAVID tools, encompassing molecular function (A), biological processes (B), and KEGG pathway analysis (C).

Supplementary Figure 8

qPCR validation of differentially expressed genes (DEGs) identified by transcriptomic profiling. Relative mRNA expression levels of Ccl8 , Acta1, Cxcl14 , Col6a3, and Aldh3b2 in the cauda epididymis of WT and Lyg1 KO mice treated with PBS or LPS. Actb was used as the internal reference gene, and relative expression was calculated using the 2-ΔΔCT method. Data are presented as mean ± SD (n=3 per group). Statistical analysis was performed by One-Way ANOVA followed by Tukey's post-hoc test. p value less than 0.05 was considered significance
