# Supplementary material for: Lyg1 deficiency aggravated LPS-induced chronic epididymal inflammation and sperm dysfunction in mouse
Source: Front Immunol. 2025 Dec 9;16:1699581. doi: 10.3389/fimmu.2025.1699581 (PMC12722883; doi:10.3389/fimmu.2025.1699581)

Supplementary Figure 7. Bioinformatics analysis of differentially expressed genes (DEGs) in mouse cauda epididymis treated with PBS versus LPS

The bioinformatics analysis was conducted using the DAVID tools, encompassing molecular function (A), biological processes (B), and KEGG pathway analysis (C).

The bioinformatics analysis of down-regulated DEGs in the PBS-treated group of WT mice

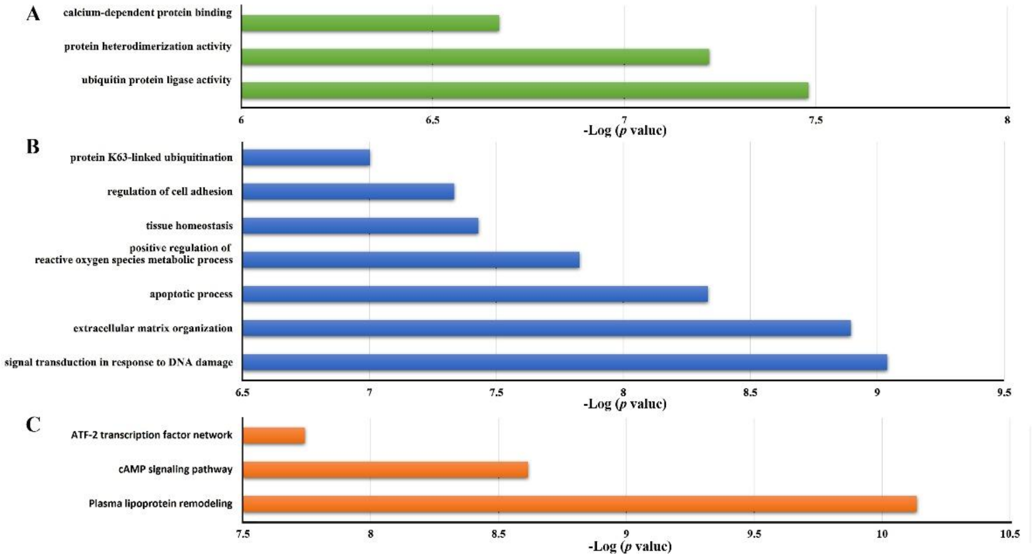

The bioinformatics analysis of up-regulated DEGs in the PBS-treated group of WT mice

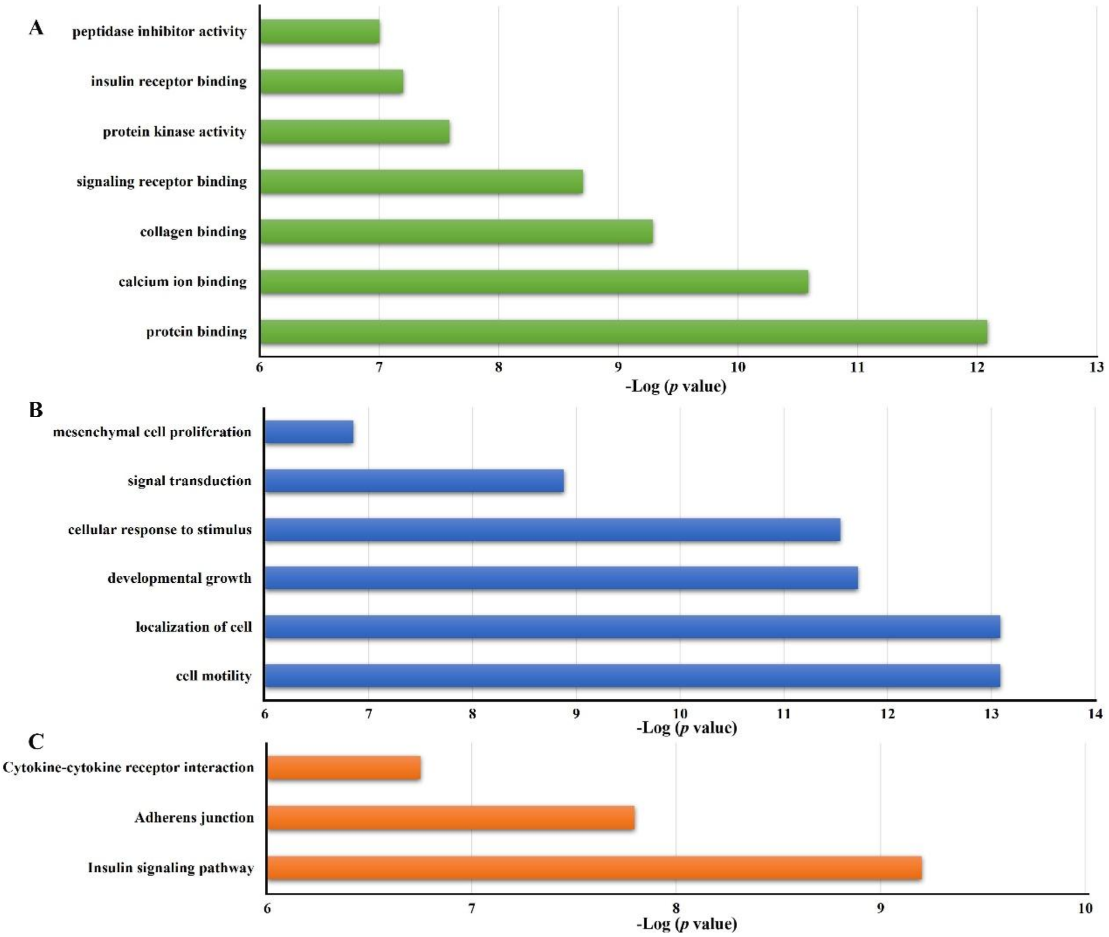

The bioinformatics analysis of down-regulated DEGs in the LPS-treated group of WT mice

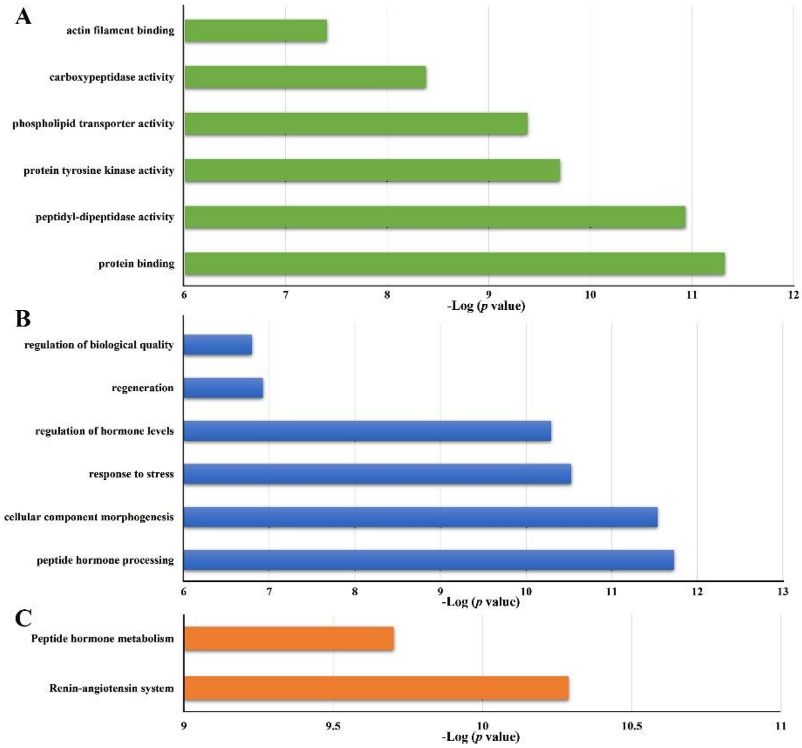

The bioinformatics analysis of up-regulated DEGs in the LPS-treated group of WT mice

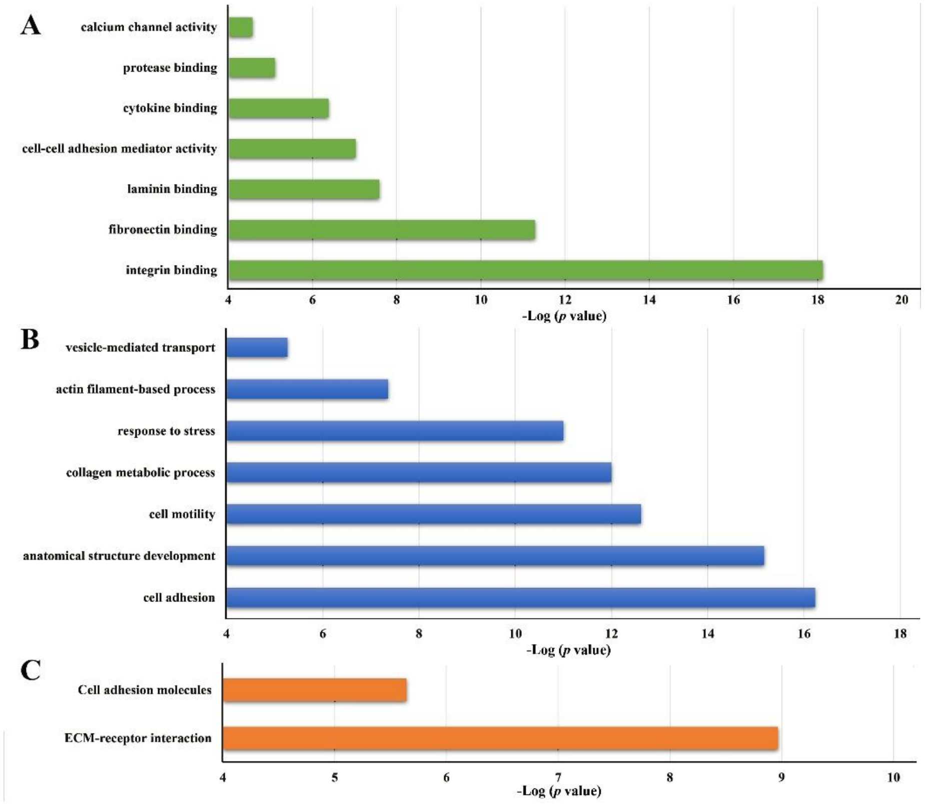

Supplement: Supplementary file 12 [file DataSheet2.pdf]
